# TLR2/MyD88 pathway-dependent regulation of dendritic cells by dengue virus promotes antibody-dependent enhancement via Th2-biased immunity

**DOI:** 10.18632/oncotarget.22525

**Published:** 2017-11-20

**Authors:** Junu Aleyas George, Seong Bum Kim, Jin Young Choi, Ajit Mahadev Patil, Ferdaus Mohd Altaf Hossain, Erdenebelig Uyangaa, Jin Hur, Sang-Youel Park, John-Hwa Lee, Koanhoi Kim, Seong Kug Eo

**Affiliations:** ^1^ College of Veterinary Medicine and Bio-Safety Research Institute, Chonbuk National University, Iksan 54596, Republic of Korea; ^2^ Department of Pharmacology, School of Medicine, Pusan National University, Yangsan 50612, Republic of Korea

**Keywords:** dengue virus, Th2-type immunity, antibody-dependent enhancement, toll-like receptor, dendritic cells

## Abstract

Possible risk mediators in primary dengue virus (DenV) infection that favor secondary DenV infection to life-threatening dengue hemorrhagic fever (DHF) and shock syndrome (DSS) via antibody-dependent enhancement (ADE) have not yet been described. Here, DenV infection enhanced the expression of inflammatory mediators and activation molecules in dendritic cells (DCs) through TLR2/MyD88 pathway. TLR2 appeared to facilitate DenV infection in DCs that were less permissive than macrophages for viral replication. In experiments using separate evaluations of DenV-infected and uninfected bystander DCs, infected DCs showed impaired maturation accompanied with TLR2-dependent production of inflammatory cytokines, by which uninfected bystander DCs showed increased expression of co-stimulatory molecules. Differential phosphorylation of MAPK and STAT3 was also detected between DenV-infected and uninfected DCs. Furthermore, DenV infection stimulated Th2-polarized humoral and cellular immunity against foreign and DenV Ag via TLR2/MyD88 pathway, and DenV-infected DCs were revealed to facilitate Th2-biased immune responses in TLR2-dependent manner. TLR2/MyD88-mediated Th2-biased Ab responses to primary DenV infection increased the infectivity of secondary homotypic or heterotypic DenV via ADE. Collectively, these results indicate that TLR2/MyD88 pathway in DC-priming receptors can drive Th2-biased immune responses during primary DenV infection, which could favor secondary DenV infection to DHF/DSS via ADE.

## INTRODUCTION

Dengue virus (DenV) is an enveloped, single-stranded, and positive-polarity RNA virus of the *Flaviviridae* family. DenV exists as four closely related serotypes (DenV1, DenV2, DenV3, and DenV4), and each causes dramatic public health problems in more than 100 countries, particularly in Asia and Latin America [1–3]. Nowadays, it is estimated that as many as 390 million people per year are exposed to DenV infection. There is a continuous increase in incidence and severity of DenV infection due to geographic expansion of its vector, the *Aedes aegypti* mosquito [1–3]. DenV infection induces diseases of varying degrees of severity in humans, from dengue fever (DF), which is usually self-limiting, to life-threatening dengue hemorrhagic fever (DHF) or dengue shock syndrome (DSS) [4]. Approximately 500,000 cases of DHF and 12,000 deaths, particularly in infants, occur in about 50-100 million cases of DF every year worldwide [5].

DHF/DSS is a severe dengue form characterized by rapid onset of capillary leak, thrombocytopenia, and altered hemostasis. Host immune responses induced by primary DenV infection have been thought to be key determinants of this severe dengue form because DHF/DSS is closely associated with heterotypic sequential DenV infection [4]. This notion suggests that DHF/DSS is a result of preexisting immune mediators not only failing to neutralize but also promoting secondary homotypic and/or heterotypic DenV infection. Antibody (Ab)-dependent enhancement (ADE) has been believed to play a crucial role in generating severe DHF/DSS at secondary DenV infection [6, 7]. ADE occurs when secondary DenV infection uses preexisting partial and/or non-neutralizing Abs induced by previous infection of the same or different serotype. Preexisting partial and/or non-neutralizing Abs can form an immune complex with DenV at the secondary infection. This complex is assumed to facilitate the infection of targets cells, including monocytes, macrophages, and mature dendritic cells (DCs), via Fcγ receptor (FcγR) [6, 7]. Using *in vitro* and *in vivo* models, many previous studies have reiterated that ADE can enhance the infection of FcγR-bearing cells, resembling that in DHF/DSS patients [8, 9]. Eventually, ADE results in higher viral load in patients, especially at early stages of infection, thereby increasing the risk of developing DHF/DSS [10, 11]. In addition, it was reported that passively transferred DenV-specific Abs in animal models resulted in evident clinical manifestation and viremia [12, 13]. These findings suggest that sub-neutralizing Abs are sufficient to favor secondary DenV infection and lead to severe DHF/DSS. Furthermore, ADE of DenV infection can reduce type I IFN production and enhance anti-inflammatory IL-10 production via defective activation of TLR and FcγR signaling pathway, thus facilitating the replication of DenV [14–16]. However, possible risk mediators in primary DenV infection that favor secondary heterotypic DenV infection to severe DHF/DSS via ADE have not yet been demonstrated.

DCs and macrophages are primary targets as well as major players in early immune responses to many viruses including DenV [17]. There is accumulated awareness that DCs can interpret pathogen-inherent signals and play a pivotal role in polarizing Th cell differentiation [18]. Recognition of pathogen-associated molecular patterns (PAMPs) by innate immune receptors such as TLR on DCs and macrophages can mediate induction of cytokines and promote Ag-presenting cell function [18]. For example, TLR1, TLR2, TLR4, TLR5, and TLR6 seem to specialize mainly in recognizing bacterial or yeast products such as lipopolysaccharide (detected by TLR4), bacterial lipoproteins, lipoteichoic acid zymosan (detected by TLR2, TLR1, and TLR6), and flagellin (detected by TLR5), while TLR3, TLR7, TLR8, and TLR9 recognize nucleic acid from pathogens, such as unmethylated CpG DNA (detected by TLR9), viral double-stranded RNA (detected by TLR3), and single-stranded RNA (detected by TLR7/8) [19]. Several viral proteins are also known to activate TLR signals, including hemagglutinin protein of measles virus [20], core protein and NS3 protein of hepatitis C virus [21], and NSP4 of rotavirus that can activate TLR2 [22], whereas respiratory syncytial virus fusion (F) protein [23], Ebola virus glycoprotein [24], and mouse mammary tumor virus envelope protein [25] induce inflammatory cytokines via TLR4 activation. In addition, it has been reported that components derived from DenV can directly activate specific TLR signals [26–28]. Notably, NS1 protein produced from DenV infection is known to activate TLR4, thereby leading to the induction and release of pro-inflammatory cytokines [26, 27]. DenV NS1 protein was also observed to activate TLR2 and TLR6 signal pathways that are involved in the immunopathogenesis of DenV infection [28]. Although the recognition of DenV infection by TLR seems to inhibit viral replication, these findings suggest that in some cases, TLR activation might induce a cellular state that favors viral replication or immunopathology such as DF and DHF/DSS. Furthermore, it has become clear that different TLR agonists can induce divergent cytokine production and signaling in DCs, subsequently inducing differentially polarized Th differentiation, which might affect disease outcomes in secondary infections [29–32]. Therefore, it is conceivable that TLR recognition of primary DenV infection in DCs might affect immune responses and immunopathogenesis induced by secondary infection by homotypic or heterotypic DenV.

Here, we sought to investigate the nature and TLR dependence of immune responses derived by DenV-infected DCs and determined their subsequent impact on secondary infection by homotypic or heterotypic DenV. Our data revealed that DenV could fully activate DCs in a TLR2/MyD88 pathway-dependent manner. Ligating TLR2 with DenV was likely to facilitate primary replication of the virus in DCs. Interestingly, DCs activated by DenV drove humoral and cellular immunity polarized to Th2-type, which might promote subsequent infection by homotypic or heterotypic DenV via ADE. These results suggest that Th2-biased Ab responses mediated by the TLR2/MyD88 pathway during primary DenV infection might favor secondary infection by heterotypic DenV and lead to a severe dengue form such as DHF/DSS.

## RESULTS

### TLR2/MyD88 pathway is required for recognition of DenV infection

**Figure 1:**
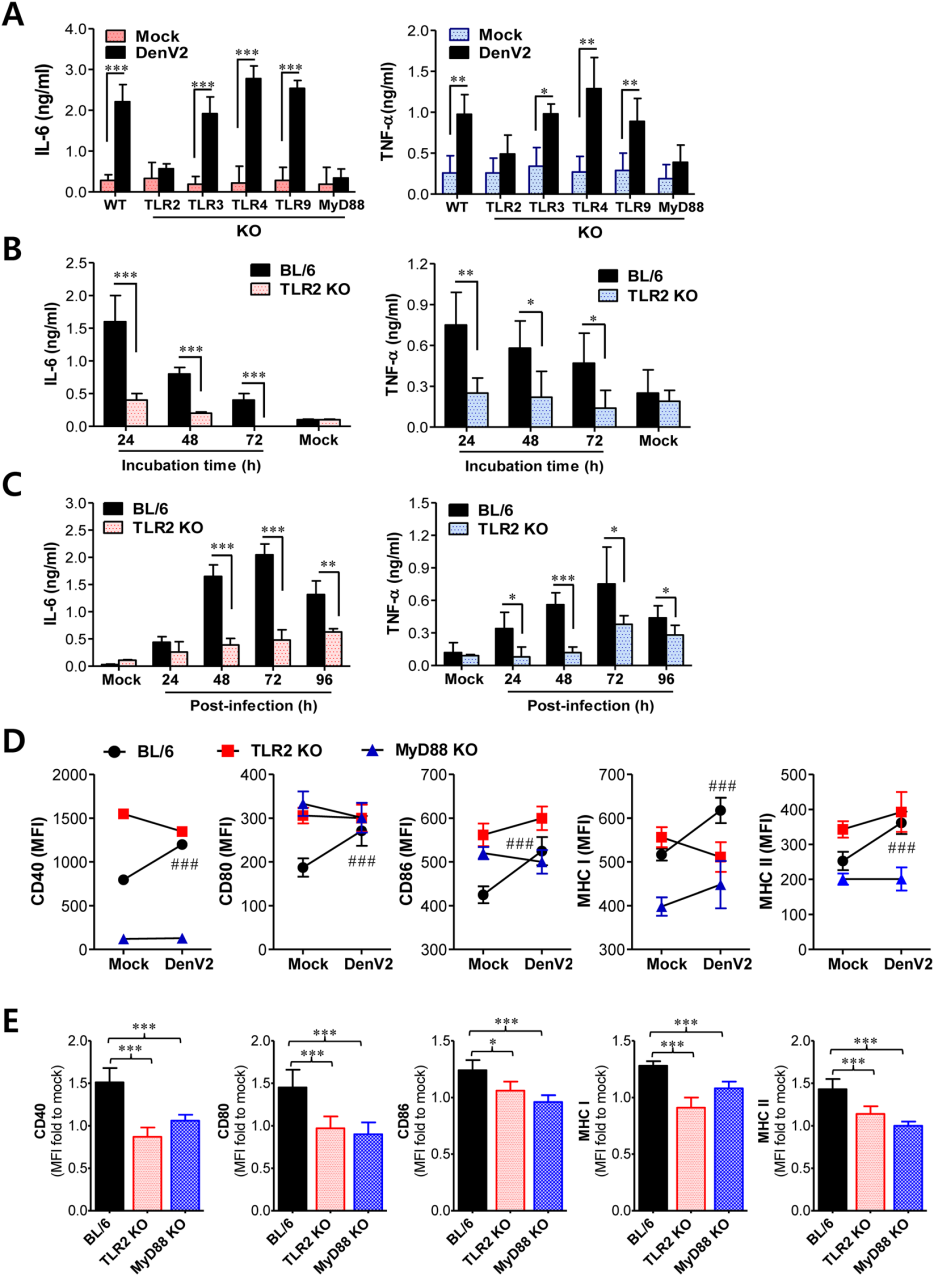
TLR2/MyD88 pathway is required for recognition of dengue virus infection **(A)** Dependence of IL-6 and TNF-α production on TLR2/MyD88 pathway in DCs infected with DenV. BMDC from wild-type BL/6, TLR2, 3, 4, 9, or MyD88 KO mice were infected with DenV2 (5.0 moi), after which cytokine levels in culture supernatants were determined by ELISA at 48 h pi. **(B)** *Ex vivo* secretion of IL-6 and TFN-α from splenocytes of BL/6 and TLR2 KO mice. Splenocytes from BL/6 and TLR2 KO mice i.p. infected with DenV2 (1×10^6^ ffu/mouse) were prepared at 48 h pi. Cytokine levels in culture supernatants were determined at indicated time after starting culture of splenocytes. **(C)** Levels of IL-6 and TNF-α in sera of BL/6 and TLR2 KO mice following DenV infection. Cytokine levels in sera collected at indicated times were determined by ELISA. **(D)** Dependence of phenotypic changes of Ag presentation-related markers on TLR2/MyD88 pathway in DenV2-infected DCs. BMDC derived from BL/6, TLR2 KO, and MyD88 KO mice were infected with DenV2 (5.0 moi) and used to stain activation markers at 24 h pi. **(E)** MFI ratio of phenotype markers to mock-infected DCs. Data denote the average ± SE of cytokines or MFI levels obtained from three independent experiments. ^*^, *p* < 0.05; ^**^, *p* < 0.01; ^***^, *p* < 0.001 compared with levels in the indicated group; ^###^, *p* < 0.001 compared with levels in mock-infected BL/6 mice.

Although it is well accepted that ADE can exacerbate disease severity in DenV infection, possible risk factors in primary infection that favor secondary infection to severe form by ADE have not yet been fully demonstrated. It has been demonstrated that intracellular signaling through TLRs can regulate host responses after various bacterial and viral infections [19]. DCs and macrophages are known as primary target cells of DenV in the host [17]. They express a wide range of innate immune receptors such as TLR that recognize pathogens [19, 32]. In particular, DCs play an important role in primary defense by generating and regulating innate and adaptive immunity upon pathogen infection. First, we examined the impact of primary DenV infection on the innate immune response of DCs and macrophages. As previously described [33], DenV2 infection induced the production of pro-inflammatory cytokines (IL-6 and TNF-α) in primary and established DCs (BMDC and DC2.4) depending on infection time and doses (Supplementary Figure 1A). However, DCs infected with DenV2 showed no detectable production of cytokine IL-10 or IL-12p70 (data not shown). The replication of infectious virus was likely to be required for inducing pro-inflammatory cytokines in infected DCs (Supplementary Figure 1B). Similarly, macrophages (BMDM, peritoneal macrophages, and RAW264.7) produced high levels of pro-inflammatory cytokines upon DenV2 infection (Supplementary Figure 1C). In order to address the impact of TLR signaling on innate responses of DCs upon primary DenV infection, we assessed the impact of each TLR molecule on primary DenV infection using BMDC derived from mice lacking a TLR such as TLR2, TLR3, TLR4, or TLR9. Among these TLR-deficient mice, only TLR2-deficient BMDC showed abrogated production of IL-6 and TNF-α in response to DenV2 infection (Figure 1A). In addition, BMDC derived from mice lacking MyD88, an adaptor molecule in signal transduction for all TLRs except TLR3, showed complete reduction of IL-6 and TNF-α following DenV2 infection. This result indicates that primary DenV2 infection in DCs can be recognized by TLR2 molecule to produce pro-inflammatory cytokines.

To further investigate the impact of the TLR2 signaling pathway on IL-6 and TNF-α production, we examined *ex vivo* and *in vivo* production of pro-inflammatory cytokines IL-6 and TNF-α following DenV2 infection. The *ex vivo* production of IL-6 and TNF-α from splenocytes of wild-type BL/6 mice infected with DenV2 was obvious compared with that in TLR2-deficient mice (Figure 1B). Consistently, DenV2 infection in wild-type BL/6 mice resulted in elevated serum levels of IL-6 and TNF-α that peaked at 72 h pi and declined thereafter, whereas TLR2-deficient mice showed marginal increase of serum IL-6 and TNF-α (Figure 1C). Taken together, these data clearly indicate that DenV2 infection is recognized by the TLR2 molecule, thereby inducing the production of pro-inflammatory cytokines. Our results are supported by a recent finding that NS1 produced by DenV2 could activate the TLR2 and TLR6 pathway [28]. Furthermore, our data are strengthened by the possibility that carbohydrate and/or lipid moiety in envelope (E) protein of DenV2 derived from mosquito cells could activate TLR2 pathway [34].

### Maturation of DCs by DenV infection depends on TLR2/MyD88 signal

Ligand binding to TLRs activates DCs, which subsequently play an important role in triggering adaptive immune responses. Thus, TLR signaling can induce maturation of DCs via up-regulating MHC and co-stimulatory molecules involved in antigen presentation to T cells [35]. Similarly, our data showed that DenV2 infection uniformly increased the expression of co-stimulatory (CD40, CD80, CD86) and MHC I/II in DCs and macrophages derived from BM cells of wild-type BL/6 mice compared with those in mock-infected DCs and macrophages (Supplementary Figure 2A). DCs and macrophages infected with DenV2 also showed higher MFI values that have usually been used for co-stimulatory and MHC I/II protein expression. Furthermore, we observed increased *in vivo* expression of co-stimulatory and MHC I/II molecules in splenic CD11c^hi^ DCs and F4/80^hi^ macrophages after mice were infected with DenV2 (Supplementary Figure 2B). This result indicates that DenV infection can induce the maturation of DCs via up-regulating co-stimulatory and MHC I/II molecules. To further ensure whether primary DenV2 infection could induce the maturation of DCs via TLR2/MyD88 signal pathway, we examined co-stimulatory and MHC I/II expression in DCs derived from TLR2- and MyD88-deficient mice upon DenV2 infection. Consistently, DCs derived from wild-type BL/6 mice showed significantly increased MFI levels in co-stimulatory and MHC I/II molecules in response to DenV2 infection, whereas DCs derived from TLR2 and MyD88-deficient mice only showed slight increase or decrease of MFI levels in these molecules (Figure 1D). When we used the ratio of MFI to mock-infected DCs to quantify changes in protein expression in co-stimulatory and MHC I/II molecules, DCs derived from TLR2 and MyD88-deificent mice showed no significant increase in MFI in these molecules compared with DCs derived from BL/6 mice (Figure 1E). These results indicate that DenV infection can induce maturation of DCs in a TLR2/MyD88 signal-dependent manner.

### TLR2 molecule facilitates infection of dengue virus in DCs

Several studies have shown that DCs and macrophages are productively infected by DenV and that this infection will lead to impaired immune responses [17, 33]. Although DCs of type I and II IFNR-deficient mice were observed to be productively infected by DenV, whether DenV can infect DCs of wild-type mice has not been fully investigated. Our data revealed that although DCs derived from wild-type BL/6 mice could be infected by DenV2, BMDC were less permissive than BMDM, with titers reaching 10-fold lower levels (Supplementary Figure 3A). The production of infectious progeny from DCs seemed to be transient, given that no progeny was detected beyond the third day (72 h) pi. Consistently, BMDC produced markedly less infectious progeny virus than BMDM (Supplementary Figure 3B). This result indicates that DenV could actively replicate in both wild-type DCs and macrophages, although DCs are less permissive of DenV replication than macrophages. When we monitored the percentage of infected cells by intracellular staining with mAb against NS1, which is produced only after productive infection, 60–70% BMDM (CD11b^+^ or F4/80^+^) were found to be positive, whereas around 20% BMDC were positive for DenV2 NS1 (Supplementary Figure 3C). To examine the *in vivo* replication of DenV in DCs and macrophages, we determined viral RNA loads in CD11c^hi^ DCs and CD11b^+^F4/80^hi^ macrophages purified from splenocytes of DenV2-infected mice. Levels of viral RNA in splenic CD11c^hi^ DCs appeared to be lower than those in splenic CD11b^+^F4/80^hi^ macrophages (Supplementary Figure 3D). We found that viral RNA loads in both splenic DCs and macrophages peaked at three days pi, after which they decreased. Collectively, our data indicate that both DCs and macrophages can be productively infected by DenV, but DCs appeared to be less permissive for replicating DenV than macrophages.

**Figure 2:**
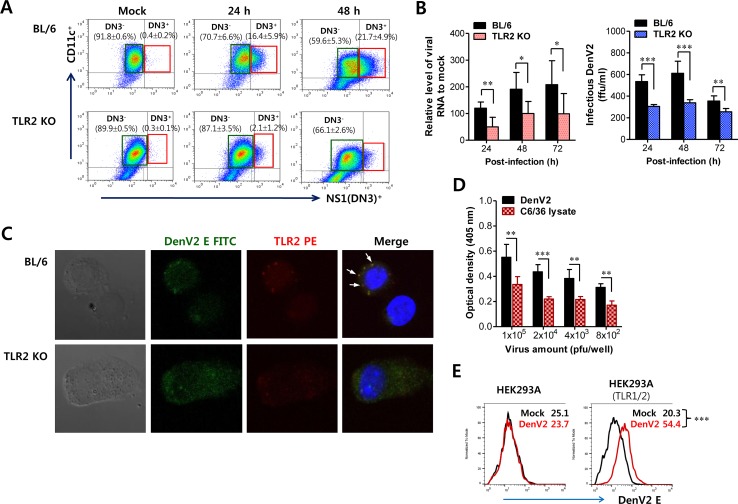
TLR2 molecule facilitates infection of DenV in DCs **(A)** Infection frequency of BMDCs derived from BL/6 and TLR2 KO mice. BMDCs derived from wild-type BL/6 and TLR2 KO mice were infected with DenV2 (5.0 moi). Infected and uninfected BMDCs were distinguished by co-staining with surface CD11c and intracellular DenV NS1 proteins at indicated time points. Values in representative dot plots denote the average percentage ± SE of indicated DN3^−^ or DN3^+^ cells in three independent experiments. **(B)** Multiplication of DenV2 in BMDCs derived from BL/6 and TLR2 KO mice. BMDCs derived from BL/6 and TLR2 KO mice were infected with DenV2 (1.0 moi). DenV2 replication was evaluated by real-time qRT-PCR and focus-forming assay using total RNA extracted from infected cells and culture supernatants, respectively. **(C)** Confocal microscopy of DenV2 Ag and TLR2 molecules in BL/6 and TLR KO BMDCs following DenV infection. BL/6 and TLR2 KO BMDCs infected with DenV2 (5.0 moi) were co-stained for DenV2 E antigen (green), TLR2 molecule (red), and nuclear stain DAPI (blue) at 24 h pi. Pictures represent at least five slides. **(D)** Binding of DenV2 virion to TLR2 molecule. DenV2 and lysate of C6/36 mosquito cells were prepared and used for examining their binding ability to TLR2 molecule. The binding ability of DenV2 to TLR2 molecules was evaluated by ELISA using mouse TLR2-fused human IgG Fc (TLR2-Fc) chimerical protein. **(E)** Cell-binding assay of DenV2 to TLR2-expressing cells. TLR1/2-expressing HEK293A cells were incubated with DenV2 for 2 h and stained with mAb specific for DenV2 E protein. HEK293A cell line was used as a negative control. Values in representative histograms denote MFI of DenV2 E protein derived from three independent experiments. ^*^, *p* < 0.05; ^**^, *p* < 0.01; ^***^, *p* < 0.001 compared with levels in the indicated group.

Cellular location of TLRs has important consequences for ligand accessibility. It can also affect downstream signaling events [19]. TLR2 is normally present at the cell surface, and TLR2-ligand complex is rapidly internalized to phagosomes or endosomes containing microbial cargo [19]. This endocytic process upon TLR2 ligation closely resembles the infection process of various viruses in host cells. Because we observed DCs to be productively infected by DenV2, we decided to explore the impact of TLR2 on the DenV2 infection process in DCs. Somewhat surprisingly, DCs derived from TLR2-deficient mice showed lower percentages of infected cells than did DCs derived from wild-type BL/6 mice (16.4% vs. 2.1% at 24 h pi and 21.7% vs. 2.8% at 48 h pi for DCs derived from wild-type or TLR2-deficient mice; Figure 2A). This result indicates that TLR2 can facilitate DenV2 infection in DCs. However, the infectivity of DenV2 in macrophages (CD11b^+^F4/80^hi^) was unlikely to change with TLR2 ablation due to high infectivity at basal level (data not shown). The lack of DenV2 infectivity on TLR2-deficient DCs was also shown by viral RNA detection using real-time qRT-PCR (Figure 2B, *left*) or focus-forming assay to measure infectious virus titer in supernatants of infected cells (Figure 2B, *right*). Also, in order to test whether TLR2 could affect the *in vivo* infectivity of DenV, we assessed DenV2 RNA load in the spleens of wild-type BL/6 and TLR2 KO mice. The TLR2 KO mice showed slightly but not significantly lower viral burden in the spleen than the wild-type BL/6 mice (Supplementary Figure 4A), which indicates that TLR2 molecule does not apparently affect *in vivo* DenV2 infection. However, splenic CD11c^hi^ DCs of TLR2 KO mice contained temporally lower viral RNA loads than those of wild-type BL/6 mice at 4 dpi, and F4/80^hi^ macrophages showed comparable viral RNA loads in both wild-type BL/6 and TLR2 KO mice (Supplementary Figure 4B). This result indicates that TLR2 molecule could affect the *in vivo* infectivity of DenV2 in at least DCs at temporally specific time pi.

Because TLR2 molecules are present at the cell surface, co-localization of TLR2 and DenV2 E protein at the cell surface was examined using confocal microscopy in order to confirm the interaction between DenV2 viral particle and TLR2 molecule. Our data revealed that the co-localization of DenV2 particles with TLR2 molecules was evident at the cell surface (Figure 2C). This result shows that DenV2 could physically interact with TLR2 at the cell surface, which may facilitate DenV2 infectivity in DCs. To further confirm the interaction of DenV2 particle with TLR2 molecules, we examined the interaction of DenV2 virus with TLR2 molecules by binding assay using murine TLR2-human Fc chimerical protein. We found that DenV2 obtained from mosquito cells (C6/36) interacted with TLR2 molecules because DenV2-treated wells showed higher optical densities than wells treated with C6/36 cell lysate, depending on the amount of virus added (Figure 2D). We also detected more DenV2 particles at the surfaces of TLR1/2-expressing HEK293A cells (Figure 2E). Collectively, these results show that DenV2 interacted with TLR2 molecules at the cell surfaces of DCs, thereby facilitating DenV2 infectivity.

### Bystander activation of uninfected DCs by DenV-infected DCs in a TLR2-dependent manner

**Figure 3:**
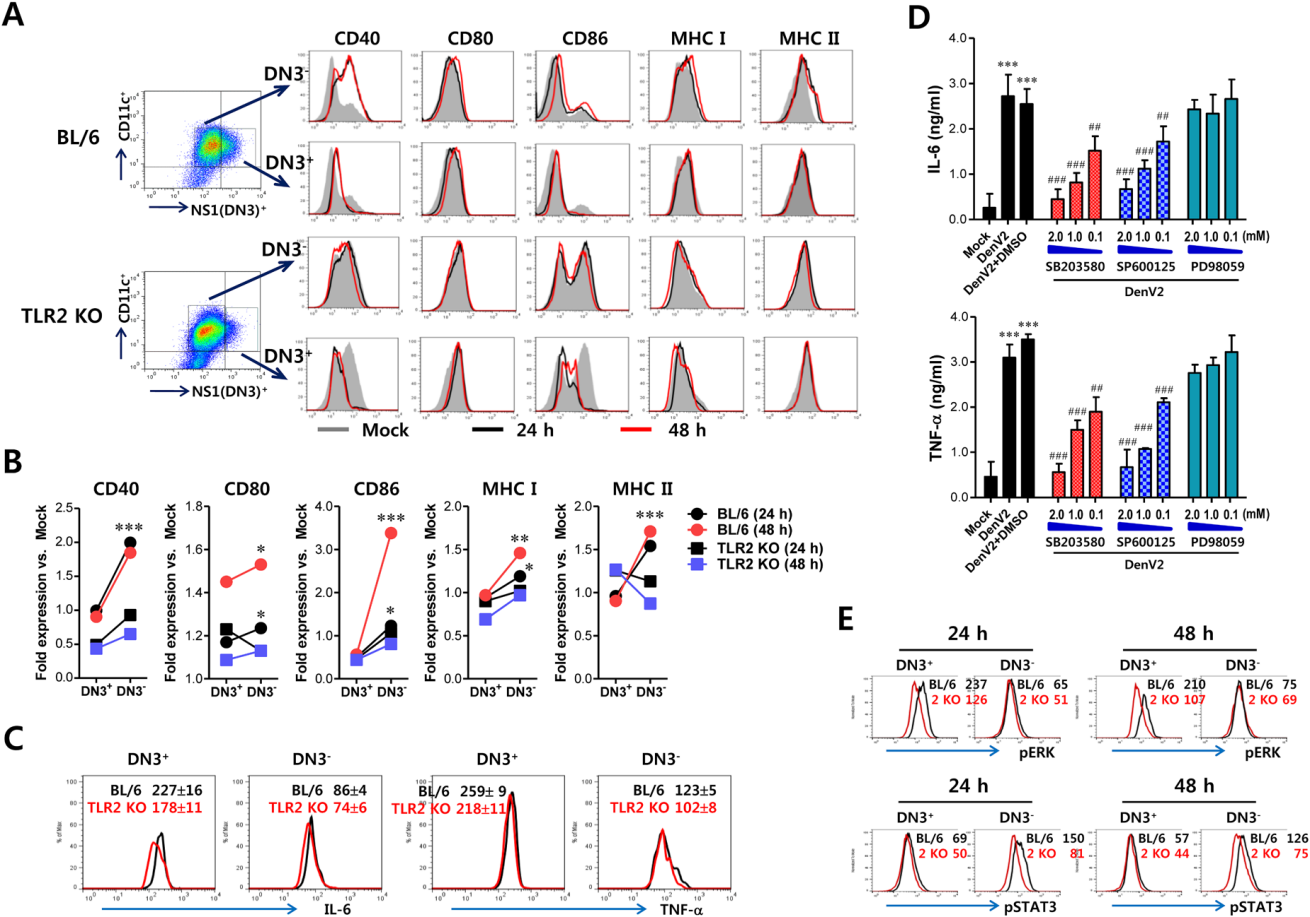
Bystander activation of uninfected DCs by DenV-infected DCs in a TLR2-dependent manner **(A)** Phenotypic levels of infected and uninfected BMDC (DN3^+^ and DN3^−^). BMDC derived from wild-type BL/6 and TLR2 KO mice were infected with DenV2 (5.0 moi) and co-stained for intracellular DenV NS1 protein and surface CD11c molecule along with phenotypic markers at 24 h (black line) and 48 h (red line) pi. Mock-infected BMDC is shown in grey. **(B)** Fold-change of phenotypic levels in infected and uninfected BMDC. Expression of each surface molecule on DN3^+^ infected and DN3^−^ uninfected DCs was quantified by MFI. Values shown in graphs represent mean fold-change in MFI vs. mock infection in three independent experiments. **(C)** TNF-α and IL-6 expression in infected and uninfected BMDCs. TNF-α and IL-6 expression by infected and uninfected BL/6 and TLR2 KO BMDC was determined by intracellular cytokine staining of DN3^+^ (infected) and DN3^−^ (uninfected) cells at 24 h pi. **(D)** Dependence of IL-6 and TNF-α production in DenV-infected DCs on p38 and JNK MAPK. BMDC derived from wild-type BL/6 were infected with DenV2 (5.0 moi) in the presence or absence of p38 inhibitor (SB203580), JNK inhibitor (SP600125), and MEK inhibitor (PD98059). Levels of TNF-α and IL-6 cytokines in culture supernatants were determined by ELISA at 48 h pi. Data represent the average ± SE of wells evaluated in quadruplicates. **(E)** Phosphorylation levels of ERK and STAT3 signal molecules in infected and uninfected BMDC. Phosphorylation level of ERK and STAT3 signal molecules was determined by intracellular staining of DN3^+^ (infected) and DN3^−^ (uninfected) cells using mAbs against phosphorylated signal molecules at 24 h pi. Values in representative histograms denote average MFI levels derived from three independent experiments. ^*^, *p* < 0.05; ^**^, *p* < 0.01; ^***^, *p* < 0.001 compared with levels in DN3^+^ cells or mock-infected group; ^##^, *p* < 0.01; ^###^, *p* < 0.001 compared with DenV2+DMSO-treated group.

Because DCs were detected with positive cells for the DenV antigen (NS1) at around 20% after infection with 5.0 MOI of virus, the majority of DCs were considered as bystander cells that might be distinct from DenV-infected DCs in functional maturation [36, 37]. To investigate the role of TLR2 molecules in functional maturation of DenV-infected and bystander DCs, we evaluated the separate maturation of DenV2-infected and uninfected bystander DCs using intracellular staining targeting DenV2 NS1. DenV2-infected DCs (DN3^+^) showed impaired maturation with lower increase in CD40, CD80/86, and MHC I/II expression. However, bystander uninfected DCs (DN3^−^) displayed matured phenotypes with significantly up-regulated cell surface expression compared with mock-infected DCs (Figure 3A). Furthermore, an interesting finding was that up-regulated expression of maturation phenotype markers (CD40, CD80/86, and MHC I/II) in bystander uninfected DCs disappeared when DCs derived from TLR2-deficient mice were infected with DenV2. In addition, the ratio of MFI to mock-infected DCs in co-stimulatory and MHC molecules was markedly higher in bystander uninfected DCs (DN3^−^) derived from wild-type mice compared with that in DenV2-infected DCs (DN3^+^; Figure 3B). However, bystander uninfected DCs derived from TLR2-deficient mice showed insignificantly increased or slightly decreased ratios of MFI to mock-infected DCs compared with DenV2-infected TLR2-deficient DCs. This result indicates that bystander uninfected DCs are relatively more activated by DenV2 infection than infected DCs, which depends on the TLR2 signal pathway.

DC maturation can be achieved by cytokine stimuli produced by DenV2 infection. DenV infection of DCs has been believed to show differential production of cytokines by virus-infected or bystander DCs [36, 37]. In a subsequent experiment, we assessed cytokines IL-6 and TNF-α expression by segregating DenV2-infected and bystander uninfected DCs with intracellular cytokine and DenV2 NS1 staining. DenV2-infected wild-type DCs showed higher production of IL-6 and TNF-α than uninfected wild-type DCs (227 ± 16 vs. 86 ± 4 for IL-6 MFI; 259 ± 9 vs. 123 ± 5 for TNF-α MFI; Figure 3C). However, IL-6 and TNF-α expression in infected wild-type DCs decreased significantly with DenV2 infection, in comparison with infected TLR2-deficient DCs (227 ± 16 vs. 178 ± 11 for IL-6 MFI in BL/6 and TLR2 KO; 259 ± 9 vs. 218 ± 11 for TNF-α MFI in BL/6 and TLR2 KO). Uninfected TLR2-deficient DCs showed slightly lower IL-6 and TNF-α expression than uninfected wild-type DCs, even though uninfected DCs usually produced IL-6 and TNF-α at low levels. These data imply that IL-6 and TNF-α are predominantly produced by virus-infected DCs rather than uninfected DCs, in a TLR2-dependent manner.

### Differential activation of MAPK and STAT3 in bystander uninfected and infected DCs

DC activation in response to many stimuli through TLRs will activate intracellular signaling transducers such as MAPKs and subsequent NF-κB [38]. Our results showed that DenV2 infection in DCs activated all MAPKs, including JNK, ERK1/2, and p38, as shown by increased levels of phosphorylated p38, ERK1/2, and JNK (Supplementary Figure 5). The p38 MAPK activation in DCs following DenV2 infection started at 2 h pi and peaked at 4–8 h pi and then declined. However, ERK1/2 phosphorylation peaked at 2–4 h pi followed by a gradual decline, and JNK and Akt showed delayed activation with the levels peaked at around 48 h pi. Multiple MAPK activation in DCs following DenV2 was revealed to facilitate I-κBα degradation at around 48 h pi, which indicates that DenV2 infection activates NF-κB. These results suggest that DenV infection can activate NF-κB and MAPKs in DCs, presumably thereby producing vast amounts of IL-6 and TNF-α. To further evaluate the effect of MAPK activation on IL-6 and TNF-α production in DenV2-infected DCs, we determined the production of IL-6 and TNF-α in the presence or absence of several MAPK inhibitors such as SB203580 (p38 inhibitor), SP600125 (JNK/SAPK inhibitor), and PD98059 (MEK inhibitor). Our data revealed that inhibition of p38 and JNK/SAPK MAPKs significantly reduced IL-6 and TNF-α production in a dose-dependent manner (Figure 3D). Of note, the reduced IL-6 production from DenV2-infected DCs was more apparent with the addition of p38 and JNK/SAPK inhibitors than was the reduced TNF-α production. Because IL-6 produced from DenV2-infected DCs binds to IL-6R on bystander uninfected DCs and subsequently activates STAT3 for intracellular signal transfer, we were interested in the differential activation of MAPK in infected DCs through TLR2 and of STAT3 in bystander uninfected DCs through IL-6R. As expected, we detected phosphorylated ERK in wild-type DCs infected with DenV2 (DN3^+^) at apparently higher levels than those in TLR2-deficient DCs (Figure 3E). However, bystander uninfected DCs (DN3^−^) derived from both wild-type BL/6 and TLR2-deficient mice showed no significant change in ERK phosphorylation level. This indicates that ERK phosphorylation occurs only in infected DCs in a TLR2-dependent manner. In contrast, STAT3 phosphorylation was more apparently induced in bystander uninfected DN3^−^ DCs than in DenV2-infected DN3^+^ DCs. Of note, TLR2-deficient DCs showed lower STAT3 activation in both bystander uninfected and infected cells compared with wild-type DCs. Collectively, these results suggest that MAPK activation through TLR2 ligation with DenV particles is achieved in infected DCs to produce IL-6, which in turn activates STAT3 in both infected and uninfected bystander DCs in autocrine and paracrine manners, respectively.

### DenV induces Ag-specific Th2 immune responses via TLR2/MyD88 pathway

**Figure 4:**
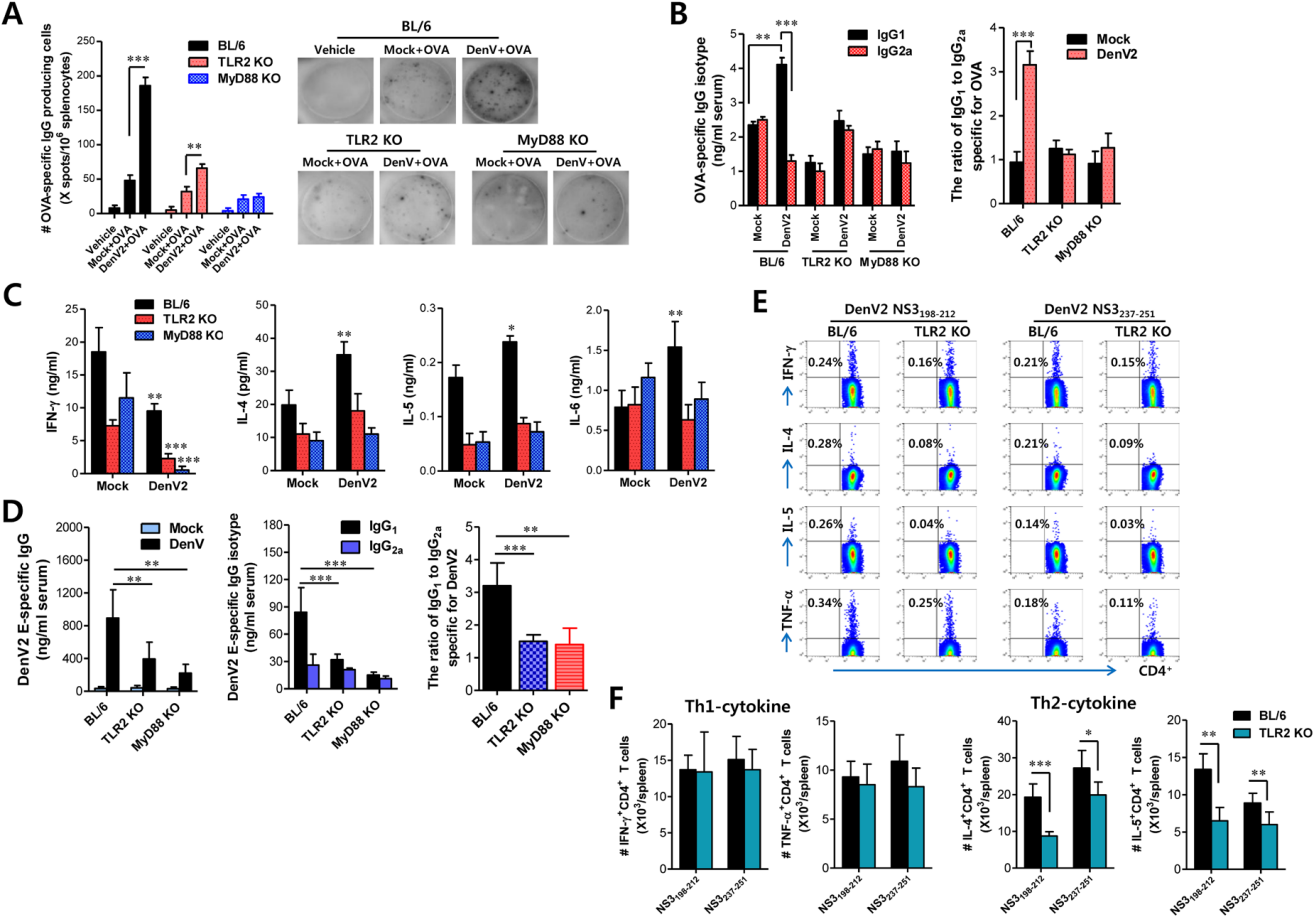
DenV induces Ag-specific Th2 immune responses in TLR2/MyD88-dependent manner **(A)** Enhanced number of anti-OVA-IgG producing cells by re-stimulation of Ag in mice infected with DenV2. Splenocytes of wild-type BL/6, TLR2 KO, and MyD88 KO mice immunized i.p. with PBS (vehicle), OVA (50 μg), or OVA (50 μg) plus DenV2 infection (1×10^6^ ffu/mouse) were prepared at 14 days post-immunization and used to enumerate anti-OVA-IgG producing cells using ELISPOT assay. Bars denote the average ± SE of the number of anti-OVA-IgG producing cells in million splenocytes obtained from four mice per group. Pictures are representative of ELISPOT in each group. **(B)** Levels and ratio of OVA-specific IgG isotypes (IgG_1_ and IgG_2a_) in sera of DenV2-infected mice. Levels of IgG isotypes in sera were determined by ELISA at 14 days after mice were immunized with PBS, OVA, or OVA plus DenV2 infection. **(C)** Prominent production of Th2-type cytokines in splenocyte of DenV2-infected mice after re-stimulation with OVA protein. Splenocytes of BL/6, TLR2 KO, and MyD88 KO mice immunized with PBS, OVA, or OVA plus DenV2 infection were prepared at 14 dpi and re-stimulated with OVA protein (100 μg/ml) for 72 h. Th1/Th2-cytokine levels in culture supernatants of re-stimulated splenocytes were determined by sandwich ELISA. **(D)** DenV2 E-specific IgG levels and its isotype ratio in DenV-infected mice. BL/6, TLR2 KO, and MyD88 KO mice were infected i.p. with DenV2 (1×10^6^ ffu/mouse) and boosted 7 days later. Levels of DenV2 E protein-specific IgG and its isotypes (IgG_1_ and IgG_2a_) were determined by ELISA at 14 days after boosting immunization. **(E, F)** Th2-biased CD4^+^ T cell responses specific for DenV Ag in TLR2-dependent manner. Splenocytes of BL/6 and TLR2 KO mice infected with DenV2 were prepared at 14 dpi and stimulated with two CD4^+^ T-cell epitope peptides (NS3_198-212_ and NS3_237-251_) for 12 h. The frequency **(E)** and total absolute number **(F)** of CD4^+^ T cells producing Th1 (IFN-γ and TNF-α) and Th2 (IL-4 and IL-5) were evaluated by co-staining with intracellular cytokines and surface CD4 molecules. Dot plots are representatives of at least three independent experiments. Bars denote the average ± SE of levels derived from at least three independent experiments (*n* = 3-4). ^*^, *p* < 0.05; ^**^, *p* < 0.01; ^***^, *p* < 0.001 compared with corresponding mock-infected or the indicated group.

There is accumulated awareness that DCs can interpret pathogen-inherent signals and play a pivotal role in polarizing Th cell differentiation [29–32]. TLR-activated DCs generally favor the development of Th1 responses due to IL-12 production by TLR ligation. However, a subset of TLR ligands appears to favor the development of Th2 responses possibly as a result of failure in IL-12 production [39]. Furthermore, it has been demonstrated that TLR2 activation facilitates different outcomes depending on the types of responding cells. For example, an endogenous TLR2 agonist was observed to skew Th responses to Th2 pattern [40], whereas triggering TLR2 on B cells was found to inhibit maturation of B cells [41]. Therefore, we were interested in determining whether DenV infection could modulate the nature of Ag-specific responses that might subsequently affect the progression of secondary infection with heterogeneous DenV infection. In order to investigate the effect of DenV infection on mounting the nature of Ag-specific adaptive immunity, we used chicken ovalbumin (OVA) to immunize wild-type BL/6, TLR2 KO, and MyD88 KO mice with foreign Ag in the presence or absence of DenV infection. Mice co-immunized with OVA plus DenV2 infection were observed to have greatly enhanced number of OVA-specific IgG-producing cells in the spleen compared with mice immunized with OVA in the absence of DenV2 infection (Figure 4A). However, the enhancement of OVA-specific IgG-producing cells was abrogated in the spleen of TLR2 KO and MyD88 KO mice following DenV2 infection. This suggests that OVA-specific IgG-producing cell enhancement by DenV2 infection depends on the TLR2/MyD88 pathway. Furthermore, assaying the subclass of anti-OVA IgG antibodies revealed that co-immunization of OVA with DenV2 infection suppressed OVA-specific IgG2a but that IgG_1_ subclass production was selectively enhanced by DenV2 infection (Figure 4B, *left*). Thus, changing the production of OVA-specific IgG isotypes by DenV2 infection greatly increased the ratio of IgG_1_ to IgG_2a_ (Figure 4B, *right*), indicating that DenV2 infection can drive immunity to be biased toward Th2-type. In contrast, significant reduction of OVA-specific IgG_2a_ isotype or selective enhancement of OVA-specific IgG_1_ isotype was not observed in TLR2- or MyD88-deficient mice. These results indicate that DenV2 infection may stimulate Th2 polarization via the TLR2/MyD88 pathway.

To determine if Ag-specific production of Th1/Th2 cytokines could be changed by DenV infection, we measured Th1/Th2 cytokine levels in culture supernatants of splenocytes stimulated with OVA protein at 14 dpi. Our data revealed that DenV2 infection enhanced the production of IL-4, a typical Th2-type cytokine, whereas the production of IFN-γ, known as Th1-type cytokine, was reduced in splenocytes of DenV2-infected mice (Figure 4C). Cytokines IL-6 and IL-5, considered to be produced by Th2-biased cells, also showed enhanced production in splenocytes of DenV2-infected mice following OVA stimulation. However, we observed marginal reduction of OVA-specific IFN-γ production in TLR2- and MyD88-deficient mice, whereas the amount of IL-4, IL-5, and IL-6 production did not change in TLR2- or MyD88-deficient mice following DenV2 infection (Figure 4C). These results indicate that the capacity of DenV infection to enhance Ag-specific Th2-polarized immune responses is dependent on the TLR2/MyD88 pathway.

To clarify that DenV infection could drive immune responses to be biased toward Th2-type, we examined adaptive immune responses specific for DenV Ag itself. TLR2- and MyD88-deficient mice displayed lower induction of IgG responses specific for DenV2 E protein compared with the IgG responses induced in wild-type BL/6 mice (Figure 4D). In particular, the production of DenV2 E protein-specific IgG_1_, but not IgG_2a_, was much more reduced in TLR2- and MyD88-deficient mice, resulting in lower ratios of IgG_1_ to IgG_2a_ than those in BL/6 mice. This implies that DenV2-specific Th2-biased immune responses following DenV2 infection depend on the TLR2/MyD88 signal pathway. Furthermore, CD4^+^ Th2 cells that produce IL-4 and IL-5 in response to stimulation with CD4^+^ T-cell epitope peptides (NS3_198-212_ and NS3_237-251_) were detected in TLR2- and MyD88-deficient mice with lower frequency than in BL/6 mice. However, IFN-γ-producing CD4^+^ Th1 cells did not change in TLR2- or MyD88-deficient mice (Figure 4E). The lower frequency of IL-4 and IL-5-producing CD4^+^ Th2 cells in TLR2- and MyD88-deficient mice resulted in significantly fewer IL-4 and IL-5-producing CD4^+^ Th2 cells in the spleen of TLR2- and MyD88-deficient mice than in BL/6 mice (Figure 4F). Taken together, these results indicate that DenV infection could induce Th2-biased immune responses against foreign Ag as well as DenV2 Ag itself in a TLR2/MyD88-dependent manner.

### DenV-infected DCs mount Ag-specific Th2 immune responses in a TLR2-dependent manner

**Figure 5:**
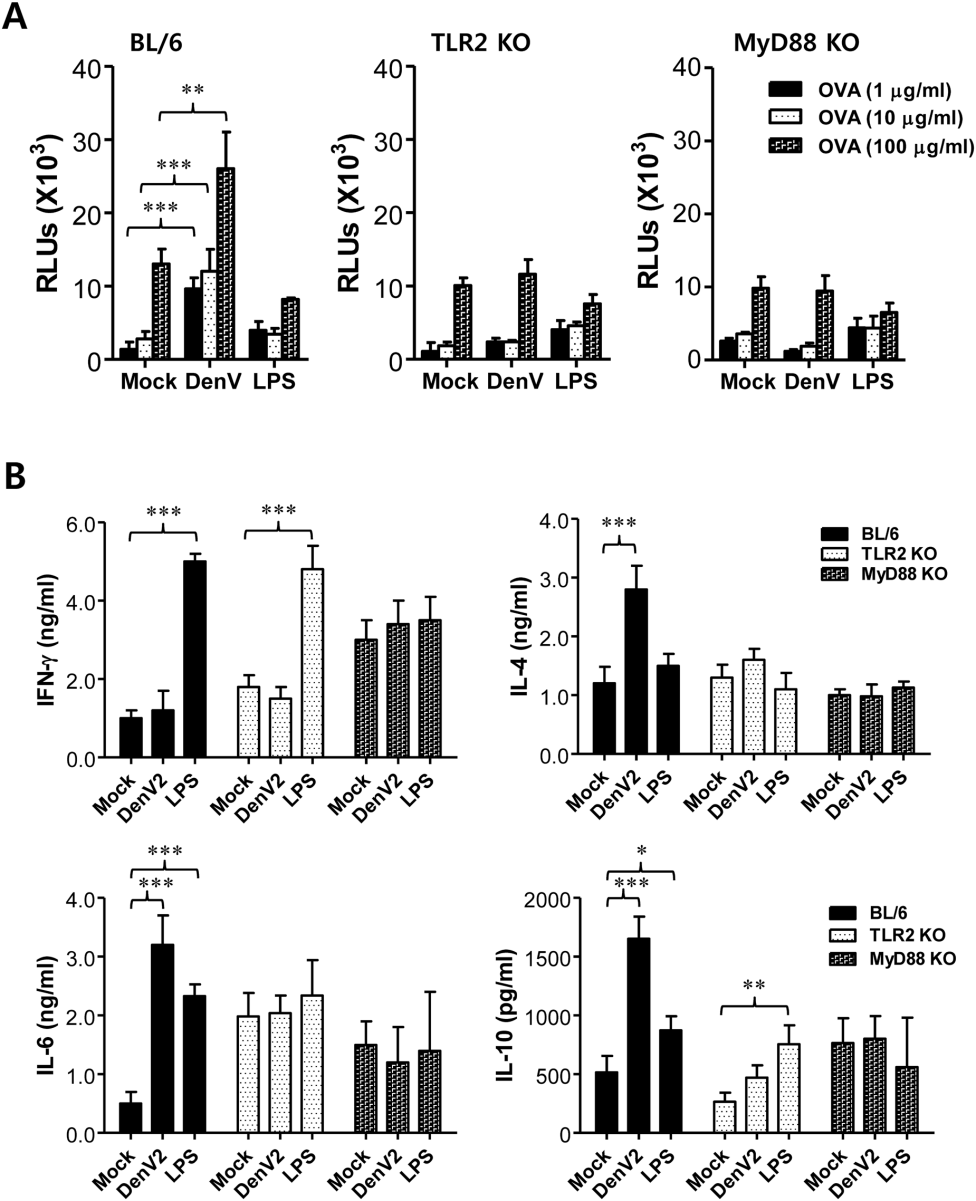
DenV-infected DCs promote Th2 immune response via TLR2/MyD88-dependent pathway **(A)** Ag-specific proliferation of splenocytes obtained from mice immunized with DenV2-infected DCs. BMDCs generated from wild-type BL/6, TLR2 KO, and MyD88 KO mice were incubated with OVA protein (100 μg/ml) for 24 h and then cultured in the presence or absence of DenV2 (5.0 moi) or LPS (1 μg/ml) for another 24 h. After thorough washing, DCs were injected i.p. to BL/6 mice (0.2 ml, 5×10^5^ cells per mouse) on day 0, immunized i.p. with OVA (50 μg/ml/0.2 ml PBS/mouse) on day 4, and killed on day 11 to prepare splenocytes. The proliferation of OVA-specific lymphocytes was evaluated by re-stimulation of splenocytes with OVA protein (1, 10, and 100 μg/ml) for 72 h. **(B)** Th1/Th2 cytokine production from splenocytes stimulated with OVA protein. Following immunization of mice with DenV2-infected DCs as described above, splenocytes were re-stimulated with OVA protein (100 μg/ml) for 72 h. Cytokine levels in culture supernatants of splenocytes were determined by ELISA. Bars show the average ± SE of cytokine levels obtained from at least three independent experiments (*n* = 3-4). ^*^, *p* < 0.05; ^**^, *p* <0.01; ^***^, *p*<0.001 compared between indicated groups.

In order to confirm the central role of DCs and the TLR2/MyD88 pathway in DenV-induced enhancement of Ag-specific Th2-polarized immune responses, we investigated whether OVA-loaded DCs infected with DenV2 could promote *in vivo* OVA-specific Th2 immune responses. To this end, we pulsed DCs derived from BL/6, TLR2 KO, and MyD88 KO mice with OVA protein, infected with DenV2 or *E. coli* LPS as a control, and injected into the peritoneal cavity of C57BL/6 mice. Mice were i.p. immunized with OVA protein on day 4 for the booster and killed on day 11 after boosting to determine splenocyte proliferation and the production of Th1/Th2-type cytokines. Our results revealed that splenocytes from mice immunized with OVA-loaded DCs in the presence of DenV2 infection proliferated in response to stimulation with OVA protein (Figure 5A, *left*), which has been known to induce the dominant proliferation of CD4^+^ Th cells [42]. Even splenocytes from mice immunized with OVA-loaded DCs infected with DenV2 showed higher proliferation upon OVA stimulation than those from mice immunized with LPS-stimulated DCs. However, the enhanced splenocyte proliferation by DenV2 infection of OVA-loaded DCs disappeared when we used DCs derived from TLR2- and MyD88-ablated mice (Figure 5A, *middle* and *right*). Moreover, splenocytes from mice immunized with OVA-loaded DCs in the presence of DenV2 infection showed higher IL-4, IL-6, and IL-10 production in response to OVA stimulation. However, there was no change in the IFN-γ production (Figure 5B). OVA-loaded DCs stimulated with LPS induced high IFN-γ production along with some IL-6 production and a marginal increase in IL-10 production. This result indicates that OVA-loaded DCs could mount Th2-biased immunity by DenV2 infection while LPS stimulation drives immune responses biased toward Th1-type. More interestingly, OVA-loaded DCs derived from TLR2- and MyD88-deficient mice showed no enhancement in the production of IL-4, IL-6, or IL-10 by DenV2 infection (Figure 5B). However, the enhanced IFN-γ production in splenocytes of mice immunized with LPS-stimulated DCs did not change in the ablation of TLR2. Taken together with the fact that DenV infection activates DCs by the TLR2/MyD88 pathway, these results suggest that DCs can promote Th2-polarized immune responses by DenV infection in a TLR2/MyD88-dependent manner.

### ADE of DenV infection is modulated by TLR2/MyD88 molecules

**Figure 6:**
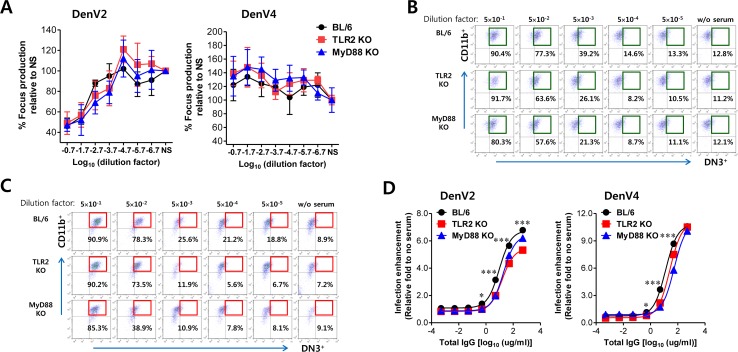
ADE of DenV infection is modulated by TLR2 and MyD88 molecules **(A)** Neutralizing activity. DenV2 or DenV4 was incubated with serially diluted sera of DenV2-infected BL/6, TLR2 KO, and MyD88 KO mice for 1 h. Titers of infectious virus in mixtures of DenV plus serum were determined by focus-forming assay in Vero cells. **(B,C)** ADE assay of DenV2 and DenV4 infection. DenV2 or DenV4 (1.0 moi) was incubated with serially diluted sera of DenV2-infected BL/6, TLR2 KO, and MyD88 KO mice for 1 h and subsequently used to infect macrophages. Macrophages infected with DenV2 (B) and DenV4 (C) were evaluated by co-staining of intracellular NS1 (DN3) and surface CD11b at 24 h pi. **(D)** Normalized fold in ADE of DenV2 and DenV4 infection by sera of DenV2-infected BL/6, TLR2 KO, and MyD88 KO mice. Values in representative dot plots denote the average percentage of infected DN3^+^ macrophages from at least four independent experiments. Data in graphs denote the average ± SE of levels derived from at least four independent experiments (*n* = 3-4). ^*^, *p* < 0.05; ^***^, *p* < 0.001 compared with levels of TLR2 KO or MyD88 KO mice.

ADE occurs when non- or partial neutralizing Abs facilitate DenV's entry into FcγR-bearing host target cells including macrophages and DCs, leading to increased infectivity in the cells at the secondary infection [6, 7]. CD4^+^ Th2 cells are believed to mediate the activation and maintenance of Ab-mediated immune responses against pathogens [43]. In the present study, because DenV2 infection drove TLR2/MyD88 pathway-dependent Th2-biased immune responses that might facilitate secondary infection with different DenV serotypes, we performed neutralization and ADE assay using Vero cells and BM cell-derived macrophages as targets, respectively. In general, anti-DenV2 sera derived from DenV2-infected BL/6, TLR2 KO, and MyD88 KO mice were unable to completely neutralize DenV2 or DenV4 infection (Figure 6A). Notably, anti-DenV2 sera obtained from BL/6, TLR2 KO, and MyD88 KO mice showed partial neutralization (between 0 and 50%) of DenV2 infection in Vero cells, whereas neutralization against infection of different serotype DenV4 was not observed. Next, we pre-incubated DenV2 and DenV4 with serially diluted anti-DenV2 sera obtained from wild-type BL/6, TLR2 KO, and MyD88 KO mice for ADE assay before we added them to FcγR-bearing cells such as macrophages. We detected infected macrophages by surface and intracellular staining against DenV NS1 glycoprotein. Enhanced infection was inversely seen with dilution factors of anti-DenV2 sera obtained from BL/6, TLR2 KO, and MyD88 KO mice (Figure 6B and 6C), which indicates that anti-DenV2 sera from BL/6, TLR2 KO, and MyD88 KO mice could increase the infectivity of DenV2 and DenV4 in FcγR-bearing macrophages. In particular, anti-DenV2 sera obtained from BL/6 mice displayed more enhanced DenV4 infectivity at serum dilutions ranging from 5×10^−4^ to 5×10^−5^ compared with DenV2 infectivity (14.6% vs. 21.2% for 5×10^−4^, 13.3% vs. 18.8% for 5×10^−5^ dilution factor in DenV2 and DenV4, respectively). However, anti-DenV2 sera obtained from TLR2 KO and MyD88 KO mice showed lower ADE of DenV2 and DenV4 infectivity than those obtained from wild-type BL/6 mice. Notably, the ADE reduction of DenV2 and DenV4 infectivity by anti-DenV2 sera obtained from TLR2 KO and MyD88 KO mice peaked at serum dilutions ranging from 5×10^−3^ to 5×10^−4^ compared with that by anti-DenV2 sera obtained from BL/6 mice (Figure 6B and 6C). This implies that ADE of anti-DenV2 sera for DenV2 and DenV4 infection in macrophages might be positively regulated by the TLR2/MyD88 pathway during primary DenV infection. The positive ADE regulation of DenV2 and DenV4 infectivity by TLR2/MyD88 pathway in primary anti-DenV2 sera was more evident when relative fold of infection enhancement to control was comparably displayed with the amount of total IgG protein (Figure 6D). Primary anti-DenV2 sera obtained from TLR2 KO and MyD88 KO mice showed lower fold of infection enhancement than those from BL/6 mice. These results suggest that Th2-biased antibody responses mediated by TLR2/MyD88 pathway during primary DenV infection are involved in ADE of secondary infection with different or same serotype DenVs, thereby leading to severe secondary DenV infections.

## DISCUSSION

DenV pathogenesis including DF and DHF/DSS has been disclosed with molecular and cellular immunological analysis; however, it remains a challenging jigsaw puzzle. Many pieces need to be uncovered to understand the complex interplay between DenV and host factors. The severity of disease outcome following DenV infection is decided by multiple potential risk factors, including the age and genetic background of patients, the serotype and genotype of DenV, and secondary infection with heterotypic DenV [4]. Mechanistic studies on DHF/DSS pathogenesis have been initially achieved with epidemiological findings. However, accumulated data using *in vitro* and *in vivo* models have contributed to the recognition of DHF/DSS mechanisms. Nowadays, important host determinants that play a role in DHF/DSS development include ADE of DenV replication [6, 7], shift of Th1 to Th2-type cytokine responses [44, 45], and other T-cell responses causing cytokine tsunami [46]. ADE theory has been the most widely accepted explanation of DHF/DSS because of the increased incidence of DHF/DSS during the first DenV infection in infants who have received maternal DenV Ab across placenta [47, 48]. These infants are predisposed to DHF/DSS when maternal DenV Ab decreases to a sub-neutralizing level before their own cell-mediated immunity is fully developed. The ADE hypothesis is also supported by increased DHF incidence associated with DenV2 infection in Cuba after prior remote infection with heterotypic DenV1 [49]. ADE theory has been further clarified by *in vivo* murine model where passively transferred DenV Ab developed evident clinical signs related to DHF/DSS and high viremia [12, 13]. However, *in vivo* and *in vitro* data in patients and animal models have not provided any host determinants in primary infection that favor secondary DenV infection to severe dengue form such as DHF/DSS. In this regard, our data presented in this study provide the first insight into the important role of Th2-biased immune responses induced by primary DenV infection and their TLR2 dependence in favoring secondary infection to DHF/DSS.

CD4^+^ Th1 cells producing IFN-γ, IL-2, and TNF-α are responsible for cell-mediated immune responses such as delayed type hypersensitivity and tissue injury in infections and autoimmune diseases, whereas CD4^+^ Th2 cells that produce IL-4, IL-5, IL-6, IL-10, and IL-13 are associated with Ab production by B cells. DCs are key players that mount Th1- and Th2-biased immune responses against Ag, but immunoregulation driven by DCs relies on the ligation of specific innate immune receptors that initiate and modulate DC maturation, resulting in the differentiation of functionally different effector DC subsets that can selectively promote Th1, Th2, Treg, or Th17 cell responses [29–32]. Our data suggest that the TLR2/MyD88 signal pathway plays a role in DC-priming receptors that drive Th2-biased immune responses during primary DenV infection. The enhancement of OVA-specific immune responses was achieved by immunizing mice either with OVA in the presence of DenV infection or with DenV-infected OVA-loaded DCs. OVA-specific immune response promoted by DenV infection is predominantly of Ag-specific Th2 type in a TLR2/MyD88-dependent manner, as corroborated by the preferential production of OVA-specific IgG_1_ as well as the generation of T cells capable of secreting elevated levels of IL-4, IL-5, and IL-6 but not IFN-γ or TNF-α. Our data further confirmed TLR2-dependent and Th2-biased immune responses against DenV Ag itself following primary DenV infection. These results are strengthened by the finding that eosinophil-derived neurotoxin, a TLR2 agonist, uses the TLR2/MyD88 signal pathway to activate DCs and enhance Ag-specific Th2-polarized immune responses in a TLR2-dependent manner [40]. Our data are also supported by several previous reports that DCs activated by TLR2 exhibit more pronounced Erk activation, produce less IL-12p70, and facilitate Ag-specific Th2-polarized immune responses [50–52]. Similarly, DCs infected with DenV showed no detectable production of IL-12p70, which might lead to a Th2-biased microenvironment. Furthermore, because DC subtypes appear to influence the nature of T-cell differentiation such as Th1 and Th2 [53], the preferential infection of specific DC subsets by DenV may promote Th cell commitment that facilitates the secondary infection with heterotypic or homotypic DenV. Therefore, characterizing major DC subsets that are infected by DenV and that promote Th2 polarization *in vivo* can contribute to uncovering more detailed role of DCs in inducing DHF/DSS in a future study. Also, all TLR2 ligands are unlikely to drive Th2-biased immune responses against certain Ag because TLR2 agonists such as Pam3, lipoteichoic acid, and macrophage-activating lipopeptide 2 are known to suppress allergic Th2 responses [54, 55]. In addition, *Propionebacterium acnes* (Pa), which activates DCs via TLR2, has been observed to induce Pa-specific Th1 immune responses [29]. Dissimilar to other TLR2 agonists, zymosan induces IL-10-producing Ag-specific Tr1 without generating Th1 or Th2 T cells [56]. Furthermore, TLR2 forms dimeric complexes with either TLR1 or TLR6 to transfer signals into cells, depending on the type of ligand [57]. The impact of TLR2 activation on the polarization of Th responses seems to be influenced by complex factors such as the type of TLR2 ligands, the means and timing of TLR2 agonist administration, and co-dependence on other PRRs. Therefore, how DenV-infected DCs drive Th2-biased immune responses in TLR2-dependent manner merits further investigation.

Nevertheless, our data provide a plausible clue to understanding the DHF/DSS pathogenesis via TLR2-dependent ADE. Serum Th1-type cytokine IFN-γ and IL-2 levels are the highest in DF patients, while serum Th2-type cytokines IL-4, IL-6, and IL-10 levels are at the maximum in the most severe cases of DHF [45]. This supports that a shift to Th2-biased immune responses via TLR2 during primary DenV infection might be an important determinant in DHF patients. Humoral immune responses promoted by CD4^+^ Th2 cells during primary DenV infection in a TLR2/MyD88-dependent manner are assumed to increase the output of secondary DenV infection. In some cases, it approached 100- to 1,000-fold [58, 59]. This ADE of secondary DenV infection resulting from avid attachment of immune complexes to FcγR-bearing cells may yield a large number of infected cells in the presence of antibodies compared with that in the absence of antibodies. This theoretical notion is based on the fact that DHF patients have higher viral burden, thereby leading to greater host inflammatory responses and increased plasma levels of pro-inflammatory cytokines [46]. Furthermore, ADE of secondary DenV infection can be amplified by enhanced expression of mannose-binding receptor (MR) in macrophages exposed to IL-4 produced from Th2-biased T cells [60], impaired type I IFN production [15, 16], and IL-10 production resulting from immune complex binding with FcγR in host cells [14]. In addition, shift to certain DenV-specific Ig subclasses including IgG_1_ with the help of CD4^+^ Th2-type cells may contribute to ADE and/or complement activation [61]. Ultimately, a higher viral burden during secondary DenV infection via TLR2-dependent ADE likely drives more production of viral components such as NS1 that can stimulate innate receptors in host cells [26, 27], thereby leading to cytokine storm in DHF patients. However, our results revealed that ADE of heterotypic or homotypic DenV infection by sera derived from wild-type BL/6, TLR2 KO, and MyD88 KO mice was evident with small differences compared with Th2 polarization and virus-specific IgG_1_ production. Conceivably, this may be driven by other mechanisms including the difference in overall immune responses against DenV infection in wild-type BL/6 and TLR2 KO mice. Indeed, TLR2 KO mice displayed modestly increased CD8^+^ T cells response specific to DenV2 Ag compared with wild-type BL/6 mice (data not shown). Thus, this contrast regulation in various parameters of immune responses against primary DenV infection may make TLR2′s role difficult to interpret in DHF/DSS via ADE.

Virus binding with host cells through cognate receptors is not only a first and critical step in viral replication but also a key factor to understand virus pathogenesis. It is already known that DenV uses DC-SIGN and MR on DCs and macrophages for its entry [60, 62]. CLEC5A is considered to transfer signals into macrophages as a signaling receptor of DenV [63]. These receptors interact with glycans located on the envelope (E) protein of DenV [64]. However, treatment with single Ab to either DC-SIGN or MR resulted in incomplete inhibition of DenV infection, even though the combination of anti-DC-SIGN and anti-MR antibodies is more effective in inhibiting DenV infection [64]. These findings indicate that other unknown entry molecules are potentially involved in infecting DCs and macrophages. Our data propose that TLR2 may be a potential candidate as a co-receptor for DenV to infect DCs because DCs derived from TLR2 KO mice, but not macrophages, showed less DenV infectivity than DCs from wild-type BL/6 mice. Virion particle of DenV seemed to have physical interaction with TLR2. It has been shown that a few viral components derived from DenV can bind to specific TLR molecules, such as ssRNA and dsRNA for TLR3 and TLR7 [65, 66] and glycosylated NS1 for TLR2 and TLR4 [26–28]. Because those viral components are not involved in the constitution of DenV particles, it is unlikely that such TLR agonists facilitate the entry of virion particles into DCs. However, interaction of DenV particles with TLR2 is supported by the finding that DenV E lipoprotein containing an unsaturated fatty acid can stimulate the TLR2-signaling pathway [34]. TLR2 ligands include various molecules with diacyl and triacylglycerol moieties, proteins, and polysaccharides [57]. TLR2 has a hydrophobic pocket that can interact with lipid chains in a nonspecific manner, allowing variations in length and chemical structure of lipid or hydrophobic moieties of its ligands [57]. Indeed, similar to our data, it has been found that TLR2 molecules can be stimulated with capsid particles of hepatitis B virus, the constituent of virion [67]. Therefore, it is conceivable that certain moieties of glycan in the envelope surface of DenV cultured in mosquito cells can bind to TLR2 molecules. This speculation suggests that TLR2 molecules are involved in the initial entry of DenV into DCs as auxiliary receptor, after which certain viral components produced by DenV replication such as NS1 could amplify the activation of TLR2 signals.

Although DCs were less permissive than macrophages for DenV replication, DCs were uniformly activated by DenV infection in our data [33]. DenV-infected DCs showed enhanced secretion of inflammatory mediators and increased expression of cell-surface molecules in a TLR2/MyD88-dependent manner. DCs and macrophages are primary target cells of DenV infection. Notably, DCs are key players that initiate immune responses and mount their nature against specific Ag. Consistent with previous reports [33], DenV-infected DCs produced significant amounts of IL-6 and TNF-a but not IL-12p70 or IL-10. These differential microenvironments orchestrated by DenV-infected DCs likely drove Th2-biased immune responses against Ag. However, detailed investigation is needed to find crucial determinants. Here, one interesting observation was that there were TLR2-dependent differences in innate responses between infected and uninfected bystander DCs in separate evaluations for maturation and cytokine profiles. DenV-infected NS1^+^ DCs showed impaired maturation, whereas uninfected bystander NS1^−^ DCs showed TLR2-dependent, higher expression of co-stimulatory molecules CD40, CD80, and CD86, as well as MHC I and II. IL-6 and TNF-α were dominantly produced from infected NS1^+^ DCs, which might affect the maturation of infected and uninfected DCs in autocrine and paracrine manners. This observation is consistent with previous results demonstrating that DenV-infected DCs produce pro-inflammatory cytokines along with impaired maturation phenotypes [36, 37]. Notably, DC exposure to TNF-α has induced the up-regulation of CD80 and CD86 in uninfected bystander cells [36]. Our data were further strengthened by differential phosphorylation of MAPK and STAT3 in DenV-infected and uninfected bystander DCs, respectively. Also, increased apoptosis of DenV-infected DCs may be able to serve as maturation stimuli for uninfected bystander DCs [36]. Indeed, engulfment of infected and apoptosized DCs by bystander DCs has been demonstrated in various infection models, such as canarypox [68], measles [69], and human immunodeficiency virus [70]. Because apoptotic bodies can serve as sources of Ag presented by uninfected bystander DCs [71], cross-presentation of DenV Ag derived from infected DCs by bystander DCs may provide an alternative route for initiating Th2-biased immune responses.

Non-neutralizing Abs against prM protein of immature DenV seem to be important in ADE because immature viruses that would be noninfectious or less infectious are rendered infectious when they combine with anti-prM [72, 73]. However, severe dengue form can also be achieved when calculated neutralizing Ab titers against secondary heterotypic virus are more than 1:100 [74]. Similarly, children with detectable neutralizing Ab in sera can develop DHF during secondary DenV infection [75]. These studies indicate that *in vitro* neutralizing activity may not be a consistent correlate of disease outcome. Our results revealed that sera derived from DenV-infected BL/6, TLR2 KO, and MyD88 KO mice showed incomplete and comparable neutralizing activity against homotypic DenV2 and heterotypic DenV4; however, these sera displayed differences in ADE. Anti-DenV2 sera derived from wild-type BL/6 displayed higher ADE than those derived from TLR2 KO or MyD88 KO mice. We propose that different usage of DenV-specific Ig subclasses for FcγR is likely to cause higher ADE in sera derived from BL/6 mice [61] due to TLR2-dependent Th2-biased responses in BL/6 mice. Understanding the detailed pathogenesis of DHF/DSS via ADE is important for developing therapeutic strategies and vaccines, and identifying conditions that favor secondary DenV infection to ADE would contribute to this development. Our results provide valuable insight into TLR2-dependent Th2-biased immune responses against primary DenV infection that facilitates secondary DenV infection via ADE.

## MATERIALS AND METHODS

### Ethics statement

All animal experiments described in the present study were conducted at Chonbuk National University according to the guidelines set by the Institutional Animal Care and Use Committees (IACUC) of Chonbuk National University and pre-approved by the Ethical Committee for Animal Experiments of Chonbuk National University (Permission code 2013-0028). Animal research protocol used in this study followed the guidelines set by the nationally recognized Korea Association for Laboratory Animal Sciences (KALAS). All experimental protocols requiring biosafety were approved by Institutional Biosafety Committees (IBC) of Chonbuk National University.

### Animals, cells, and viruses

C57BL/6 mice (BL/6, H-2^b^) of 5 to 6 weeks old were purchased from Samtako (O-San, Korea). TLR2, TLR3, TLR4, TLR9, and MyD88-deficient mice (H-2^b^ genetic background) generated by S. Akira (Osaka University, Osaka, Japan) were kindly provided by the Immunoregulatory Research Center (Ulsan, Korea) and International Vaccine Institute (Seoul, Korea). DenV serotype 2 (DenV2) strain DJB2001 and serotype 4 (DenV4) strain DJB4002 are Vietnamese isolates obtained from Yong-Seok Jang, Chonbuk National University, Korea [76, 77]. Following four serial passages in mouse brain, the viruses were propagated in mosquito *Aedes albopictus* cell line C6/36 (American Type Culture Collection) using DMEM supplemented with 2% fetal bovine serum (FBS), penicillin (100 U/ml), and streptomycin (100 U/ml). C6/36 cultures were infected with either DenV2 or DenV4 at a multiplicity of infection (moi) of 0.1 and incubated in a humidified CO_2_ incubator at 28°C for 1 h. After absorption, the inoculum was removed and 7 ml of a maintenance medium containing 2% FBS was added. At approximately 6–7 days post-infection (dpi), cultures of host cells showing 80–90% cytopathic effect were harvested. Virus stocks were titrated by focus-forming assay using DenV NS1-specific monoclonal antibody (DN3; Abcam, Cambridge, UK), and stored in aliquots at −80°C until use.

### Antibodies and reagents

The following mAbs were obtained from eBiosciences or BD Biosciences (San Diego, CA, USA) for FACS analysis and other experiments: fluorescein isothiocyanate (FITC)-anti CD40 (3/23), CD80 (16-10A1), CD86 (GL1), MHC I (28-14.8), MHC II (25-9-17), phycoerythrin (PE)-anti CD11c (M1/70), F4/80 (BM8), TLR2(CD282; 6C2), Alexa Fluor488-anti-ERK1/2 (pT202/pY204; 20A), and STAT3 (pY705; 4/P-STAT3). mAbs specific for E (clone no. DE1) and NS1 (clone no. DN3) protein of DenV were obtained from Abcam. FITC-labeled goat anti-mouse IgG was obtained from Southern Biotech (Birmingham, AL, USA). Recombinant mouse TLR2-Fc chimera, a mouse TLR2 molecule fused with human IgG_1_-Fc, was obtained from R&D Systems (Minneapolis, MN, USA). Chicken ovalbumin (grade V) and *E. coli* lipopolysaccharide (LPS) were purchased from Sigma-Aldrich (St. Louis, MO, USA). Primers specific for DenV2 E protein (Forward 5′-CAA TAT GCT GAA ACG CGA GAG AAA-3′ and Reverse 5′-AAG ACA TTG ATG GCT TTT GA-3′) were synthesized at Bioneer Corp., Daejeon, Korea.

### Preparation of BM-derived DCs and macrophages

DCs derived from BM cells (BMDC) were prepared as described earlier [78] with some modifications. Briefly, BM cells from femurs and tibiae of BL/6, TLR2 KO, and MyD88 KO mice were cultured in RPMI supplemented with 2 ng/ml GM-CSF and 10 ng/ml IL-4. On day 4, the culture was replenished with fresh media containing growth factor and cytokine. Cells were harvested on day 8 for use and then characterized by flow cytometric analysis. The culture generally consisted of > 75% CD11c^+^ cells (CD11c^+^CD11b^+^, 25%; CD11c^+^CD8α^+^, 65%). BM-derived macrophages (BMDM) were prepared by culturing BM cells in DMEM containing 30% conditioned culture media of L929 cells [79] for 7 days. The prepared BMDM was composed of > 85% F4/80^+^ cells (F4/80^+^CD11b^+^, 99.2%; F4/80^+^CD11c^+^ cells, ∼1%).

### Quantitative SYBR Green-based real-time PCR for viral replication

Levels of viral RNAs in DenV2-infected cells or tissues of DenV2-infected mice were determined by quantitative SYBR Green-based real-time RT-PCR (real-time qRT-PCR) analysis following reverse transcription of total RNA isolated from infected samples. The reaction mixture contained 2 μl of template cDNA, 10 μl of 2× Master Mix, 1.5 mM MgCl_2_, and 100 nM primers at a final volume of 20 μl. These reactions were denatured at 95°C for 10 min and then subjected to 40 cycles of 95°C for 30 s, 58°C for 30 s, and 72°C for 30 s. After the reaction cycle was completed, the temperature was increased from 65°C to 95°C at a rate of 1°C/min, and the fluorescence was measured every 15 s to construct a melting curve. A control sample containing no template DNA was run with every assay. All determinations were performed at least in duplicate in order to achieve reproducibility. The authenticity of the amplified product was determined by melting curve analysis. The ratio of viral RNA in the infected sample to uninfected sample was determined. All data were analyzed using Bio-Rad CFX Manager version 2.1 analysis software (Bio-Rad Laboratories).

### Cytokine ELISA

Sandwich ELISA was used to determine cytokine levels in culture supernatants. ELISA plates were coated with TNF-α (1F3F3D4), IL-4 (11B11), IL-5 (TRFK5), IL-6 (MP5-20F3), IL-10 (JES5-16E3), and IFN-γ (R4-6A2) antibodies (eBioscience or BD Bioscience) and incubated at 4^°^C overnight. After plates were washed three times with PBS containing 0.05% Tween 20 (PBST), they were blocked with 3% nonfat dried milk at 37^°^C for 2 h. Culture supernatant and standards for recombinant cytokine proteins (Peprotech, Rehovot, Israel) were added to these plates and incubated at 37^°^C for 2 h. Plates were washed again with PBST and biotinylated TNF-α (polyclonal antibody), IL-4 (BVD6-24G2), IL-5 (TRFK4), IL-6 (MP5-32C11), IL-10 (JES5-2A5), and IFN-γ (XMG1.2) antibodies were added. The mixture was incubated overnight at 4^°^C followed by washing with PBST and subsequent incubation with peroxidase-conjugated streptavidin (eBioscience) at 37^°^C for 1 h. Color was then developed by adding a substrate (ABTS) solution. Cytokine concentrations were determined using an automated ELISA reader and SoftMax Pro4.3 by comparison with two concentrations of standard cytokine proteins.

### Cell-binding and TLR2-binding assay

Cell-binding assay of DenV2 was performed as described previously [80]. Briefly, TLR1/2-expressing HEK293A cells (InvivoGen, San Diego, CA, USA) were incubated with DenV2 (10 moi) at room temperature for 2 h. After washing with ice-cold FACS buffer and fixed with 4% paraformaldehyde/PBS, surface-bound DenV2 was detected with mAb specific for DenV2 E protein (Abcam) and anti-mouse IgG-PE followed by FACS analysis. HEK293A cell line was used as a negative control. For TLR2-binding assay of DenV2, 96-well plates were coated with serially diluted DenV2 (100 ng/well based on protein amount) and incubated overnight at 4^°^C. Cell lysate (100 ng/well based on protein amount) was used as a negative control. After plates were washed three times with PBST and blocked with 3% nonfat dried milk for 2 h at 37^°^C, murine TLR2-human Fc chimerical protein (100 ng/well; R&D Systems) was added to these plates followed by incubation at 37^°^C for 90 min. After these plates were washed with PBST, they were incubated with anti-human IgG-HRP for 1 h followed by color development.

### Enumeration of chicken OVA-specific Ig-secreting cells by ELISPOT

Frequencies of OVA-specific IgG producing cells were determined by enzyme-linked immunospot (ELISPOT) assay. Briefly, ELISPOT plates (Millipore, Bedford, MA, USA) were coated with soluble OVA protein (200 ng/well) and incubated at 4^°^C overnight. After blocking with RPMI medium supplemented with 10% FBS, splenocytes obtained from immunized mice were seeded and incubated in triplicate at 37^°^C for 72 h. Following 72 h of incubation, plates were washed three times with PBS and three times with PBST. Biotinylated anti-mouse IgG was added to plates and incubated at 37^°^C for 1 h. Spots were developed using nitroblue tetrazolium (Sigma) and 5-bromo-4-chloro-3-indolylphosphate (Sigma) as substrate after incubation with alkaline phosphatase-conjugated streptavidin (Jackson ImmunoResearch, West Grove, PA, USA) for 1 h. They were counted 24 h later under a stereomicroscope.

### Immunization and detection of OVA-specific Igs, splenocyte proliferation, and Th1/Th2 cytokine production

BL/6, TLR2 KO, and MyD88 KO mice (*n* = 6-8) were i.p. injected with 0.2 ml PBS containing 50 μg OVA in the presence or absence of DenV (1×10^6^ ffu/mouse). At 14 days after injection, sera samples were collected to determine OVA-specific Igs. OVA-specific IgG antibodies and its isotypes were measured by ELISA as described previously [81]. Briefly, ELISA plates were coated with OVA protein (100 ng/well) and incubated at 4^°^C overnight. These plates were washed three times with PBST and blocked with 3% nonfat dried milk at 37^°^C for 2 h. Then serum samples and standard Igs were added to these plates followed by incubation at 37^°^C for 90 min. After these plates were washed with PBST, they were incubated with horseradish peroxidase-conjugated anti-mouse Igs for 1 h followed by color development.

OVA-specific splenocyte proliferation and/or Ag-specific production of Th1/Th2 cytokines were determined by stimulation of splenocytes with OVA protein as described previously [40] with minor modifications. Briefly, splenocytes of each group of mice (5×10^5^ cells/well) were prepared and seeded in triplicate into round-bottomed 96-well plates in complete RPMI 1640 medium (0.2 ml/well) and incubated in the presence or absence of OVA protein (100 μg/ml) at 37^°^C in a CO_2_ incubator for 72 h. After the 72-h incubation, replicate cultures were transferred to V-bottom 96-well culture trays and subsequently centrifuged to collect cells. Proliferated cells were then evaluated using a Vialight cell proliferation assay kit (Cambrex Bio Science, Rockland, ME, USA) according to the manufacturer's instructions. Alternatively, culture supernatants were harvested following 72-h stimulation with OVA and used to determine cytokine levels by sandwich ELISA.

### Th1-Th2 responses of DenV-specific CD4^+^ T cells

Th1-Th2 responses of CD4^+^ T cells specific for DenV were evaluated by DenV2 E-specific IgG isotypes and Th1-Th2 cytokine expression in DenV-specific CD4^+^ T cells. DenV E protein-specific IgG and its isotypes (IgG_1_ and IgG_2a_) were determined by ELISA using DenV E protein (ProSpec, East Brunswick, NJ, USA) as described above. The frequency and enumeration of Th1-Th2-biased DenV-specific CD4^+^ T cells were evaluated by intracellular Th1 (IFN-γ and TNF-α) and Th2 (IL-4 and IL-5) cytokine staining after re-stimulation of splenocytes with DenV-specific CD4^+^ T-cell epitope peptides (NS3_198-212_, GKTKRYLPAIVREAI; NS3_237-251_, GLPIRYQTPAIRAEH). Splenocytes were prepared from DenV2-infected BL/6 and TLR2 KO mice at 14 dpi and cultured in 96-well culture plates (5×10^5^ cells/well) in the presence of synthetic peptide epitopes (NS3_198-212_ and NS3_237-251_) for 12 h. Monensin at concentration of 2 μM was added to antigen-stimulated cells 6 h before harvest. These cells were washed twice with PBS and surface stained with FITC-anti-CD4 antibody at 4°C for 30 min. After the cells were washed twice with PBS containing monensin and fixation, they were washed twice with permeabilization buffer (eBioscience) and stained with PE·Cy5.5-anti-IFN-γ (XMG1.2) and APC-anti-IL-4 (BVD6-24G2) or with PE-anti-IL-5 (TRFK4) and APC-anti-TNF-α (MP6-XT22) in permeabilization buffer at room temperature for 30 min. Finally, these cells were washed twice with PBS and fixed with fixation buffer. Sample analysis was performed on a FACS Calibur flow cytometer (Becton Dickson Medical Systems, Sharon, MA, USA) using FlowJo (Tree Star, San Carlos, CA, USA) software.

### Focus reduction neutralization test

Neutralizing activities of sera derived from DenV2-infected BL/6, TLR2 KO, and MyD88 KO mice were determined by focus reduction neutralization test (FRNT) [81]. Briefly, serially diluted antibody was mixed with an equal volume of virus (DenV2, DenV4) and incubated at 37^°^C for 1 h. The mixture was then transferred to Vero cell monolayers followed by focus-forming assay.

### ADE assay of DenV infection

Serially diluted anti-DenV2 sera derived from DenV2-infected BL/6, TLR2 KO, and MyD88 KO mice based on total Igs were incubated with an equal volume of virus (1.0 moi for DenV2 and DenV4) at 37^°^C for 1 h. The solution was then transferred to macrophages and incubated at 37^°^C for 24 h. Infected macrophages were stained for surface CD11b molecule following blocking FcR with CD16/32 (eBioscience). They were then fixed and permeabilized with 4% paraformaldehyde and 0.5% saponin. DenV NS1 Ag was then stained intracellularly with anti-DenV NS1 (DN3) followed by PE-anti-mouse IgG Abs and analyzed by flow cytometry.

### Statistical analysis

All data were expressed as average ± standard error (SE). Statistical differences between groups were analyzed using unpaired two-tailed Student's *t*-test for *in vitro* experiments and immune cell analysis or ANOVA and post hoc test for multiple comparisons of means. A *p* value ≤ 0.05 was considered statistically significant. All data were analyzed using Prism software (GraphPadPrism4, San Diego, CA, USA).

## SUPPLEMENTARY MATERIALS FIGURES AND TABLES



## Abbreviations

ADE: antibody-dependent enhancement
DCs: dendritic cells
DenV: dengue virus
DHF: dengue hemorrhagic fever
DSS: dengue shock syndrome
WT: wild-type
KO: knock-out
BM: bone marrow
BMDC: BM-derived DC
BMDM: BM-derived macrophage
dpi: days post-infection
OVA: chicken ovalbumin
