# Supplementary material for: TLR2/MyD88 pathway-dependent regulation of dendritic cells by dengue virus promotes antibody-dependent enhancement via Th2-biased immunity
Source: Oncotarget. 2017 Nov 20;8(62):106050–70. doi: 10.18632/oncotarget.22525 (PMC5739701; doi:10.18632/oncotarget.22525)
Supplement: Supplementary file 1 [file oncotarget-08-106050-s001.pdf]

TLR2/MyD88 pathway-dependent regulation of dendritic cells by dengue virus promotes antibody-dependent enhancement via Th2-biased immunity

SUPPLEMENTARY MATERIALS

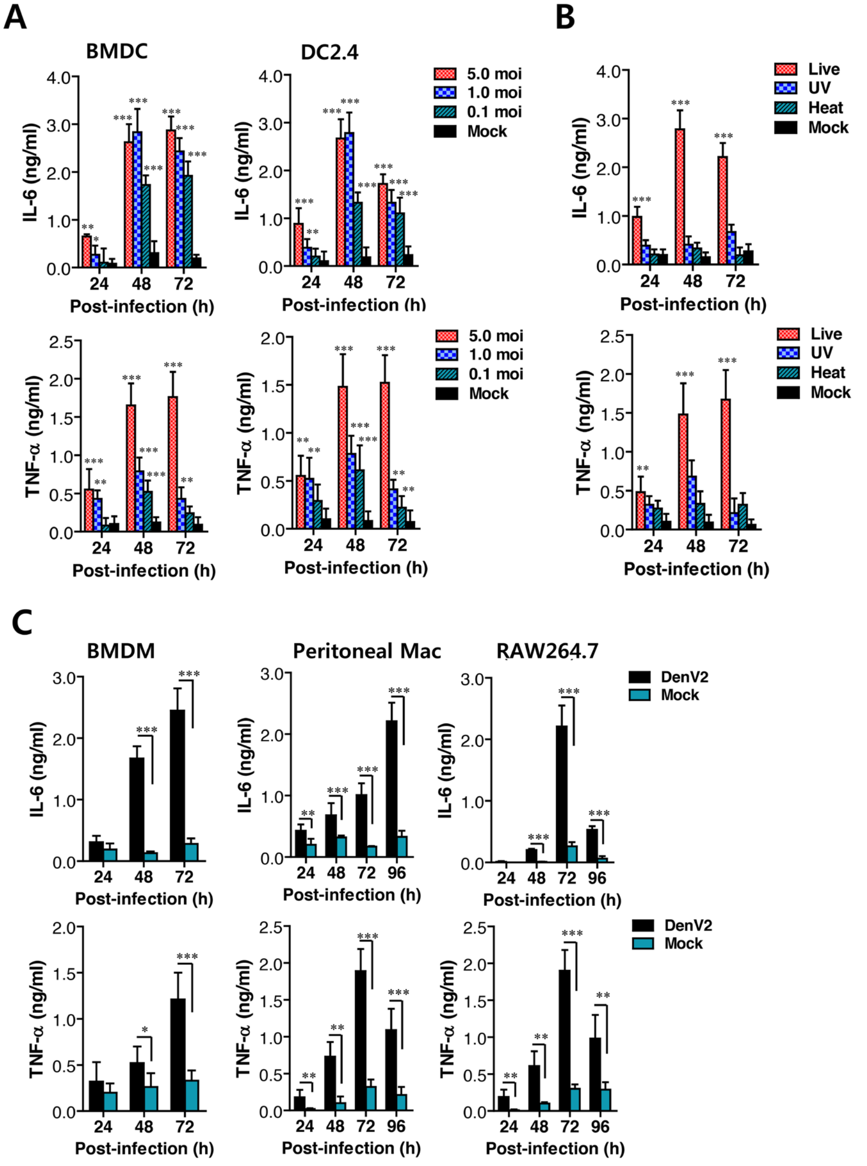

Supplementary Figure 1: Production of pro-inflammatory cytokines IL-6 and TNF-α from DCs and macrophages following DenV infection. **(A)** IL-6 and TNF-α production in DCs. Bone marrow-derived DCs (BMDCs) and established DCs (DC2.4) were infected with DenV2 (5.0, 1.0, and 0.1 moi). **(B)** Viral replication is required for the production of cytokines in DCs. DCs were infected with live DenV2 (5.0 moi) or equivalent amount of viruses inactivated by UV-irradiation (30-min irradiation) or heat (heating at 95°C for 10 min). **(C)** IL-6 and TNF-α production in macrophages. Bone marrow-derived macrophages (BMDM), peritoneal macrophages (peritoneal Mac), and established macrophages (RAW264.7) were infected with DenV2 (5.0 moi). Cytokine levels in culture supernatants were determined by sandwich ELISA at indicated time. Data in graphs denote the average ± SE of cytokine levels in quadruplicate wells of infected cells from at least three independent experiments. *, $p < 0.05$; **, $p < 0.01$; ***, $p < 0.001$ compared to levels of the indicated or mock-infected group.

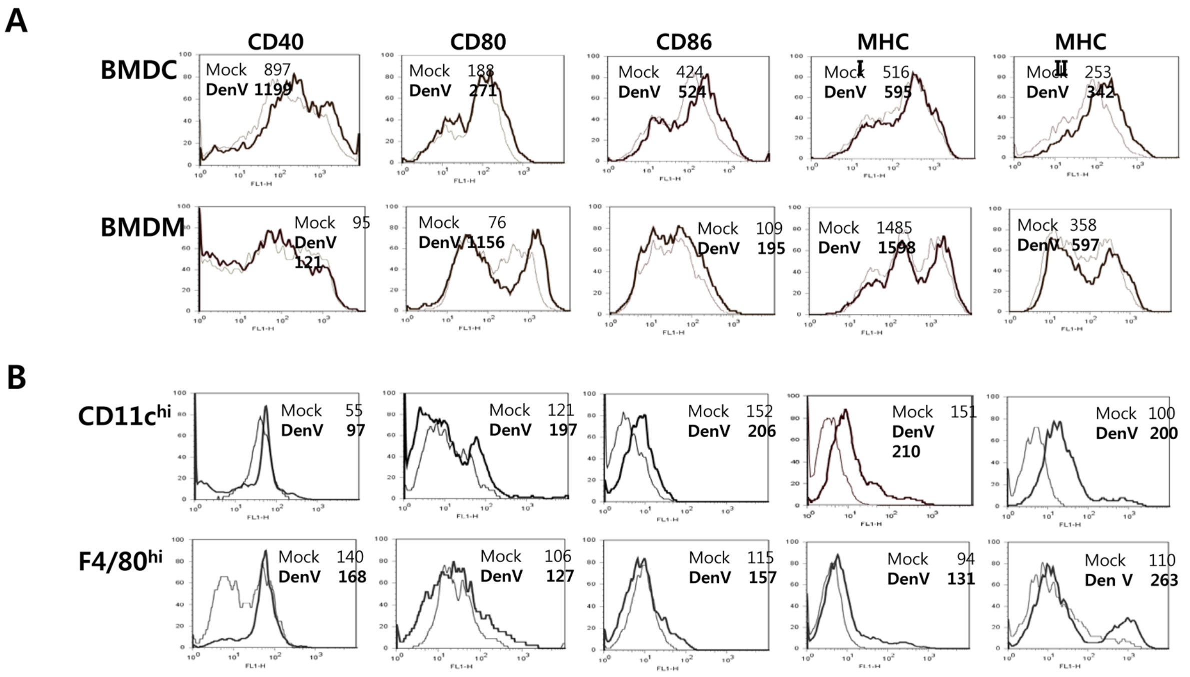

Supplementary Figure 2: Phenotypic changes of DCs and macrophages following *in vitro* and *in vivo* infection with DenV. (A) Phenotypic changes of DenV-infected BMDC and BMDM. BMDC and BMDM were infected with DenV2 (5.0 moi) and stained with corresponding antibody cocktail. **(B)** *In vivo* phenotypic levels of splenic $CD11c^{hi}$ DCs and $CD11b^{+}F4/80^{hi}$ macrophages in BL/6 mice infected with DenV. BL/6 mice were infected with DenV2 (1×10^{6} ffu/mouse) and splenocytes were prepared by digestion with collagenase at 3 dpi. Splenocytes were co-stained for surface costimulatory molecules along with CD11c, CD11b, and F4/80 molecules using antibody cocktail. Histograms show representative one of indicated costimulatory molecules on splenic DCs (gated on $CD11c^{hi}$) and macrophages (gated on $CD11b^{+}F4/80^{hi}$) from four mice per group. Values in histograms denote the average of relative MFI levels obtained from at least three independent experiments.

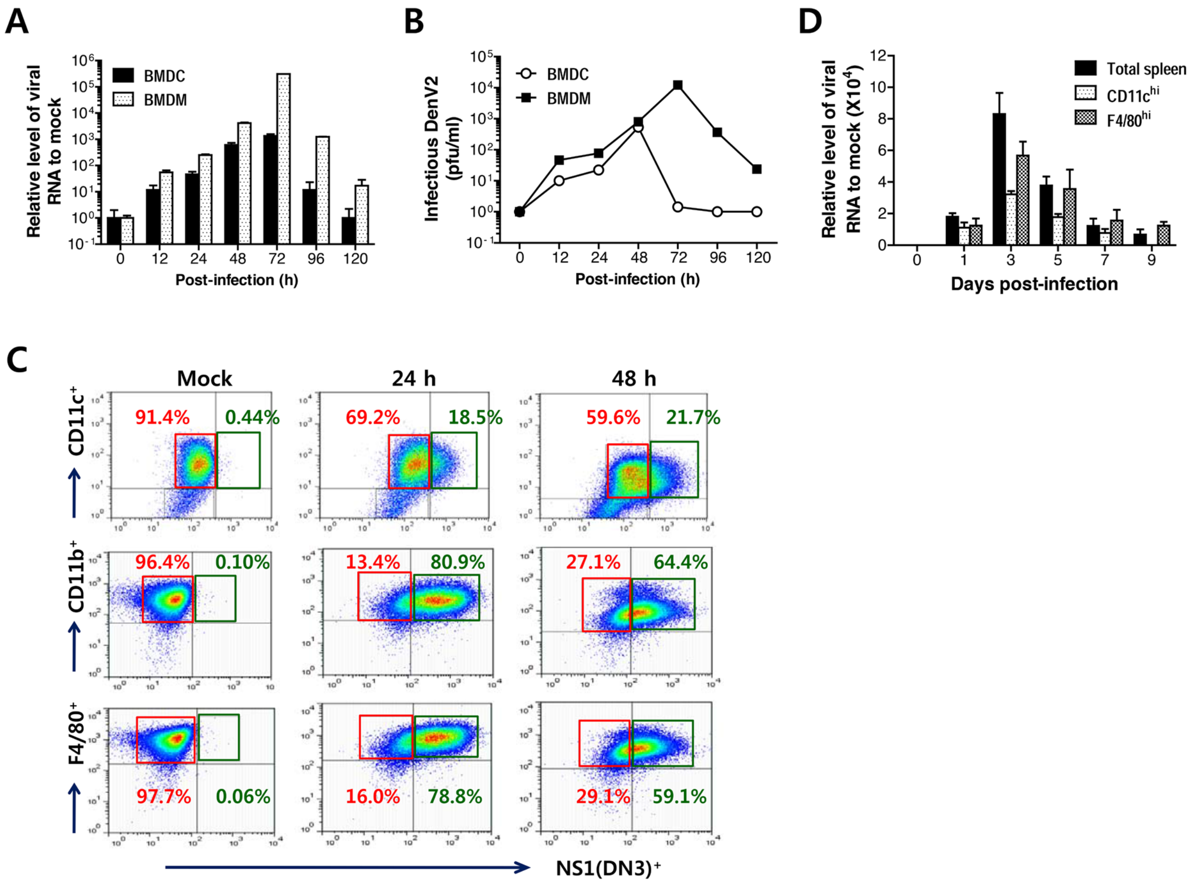

Supplementary Figure 3: *In vitro* and *in vivo* multiplication of DenV in DCs and macrophages. **(A)** *In vitro* multiplication of DenV2 in BMDC and BMDM. After infecting BMDC and BMDM with DenV2 (1.0 moi), total RNA was extracted at indicated time point and used for quantification of viral RNA by real-time qRT-PCR. **(B)** Production of infectious DenV2 from BMDC and BMDM. BMDC and BMDM were infected with DenV2 (1.0 moi) and viral titers in supernatants were determined by focus-forming assay at indicated time point. **(C)** Proportion of BMDC and BMDM infected *in vitro* with DenV2. BMDC and BMDM were infected with DenV2 (5.0 moi) and co-stained for surface molecules (CD11c, CD11b, and F4/80) and intracellular DenV NS1 protein. The proportion of infected cells was identified by intracellular DenV NS1 protein expression ($DN3^+$) as compared to mock-infected cells. **(D)** *In vivo* multiplication of DenV2 in splenic $CD11c^{hi}$ DCs and $CD11b^+F4/80^{hi}$ macrophages. $CD11c^{hi}$ and $F4/80^{hi}$ cells in DenV2-infected BL/6 mice were sorted by FACS Aria at indicated time point. Total RNAs were extracted from sorted cells and the spleen and used to determine viral RNA by real-time qRT-PCR. Levels of viral RNA were expressed as relative levels to uninfected samples after normalized with β-actin. Data in graphs denote the average ± SE of levels derived from at least four independent experiments.

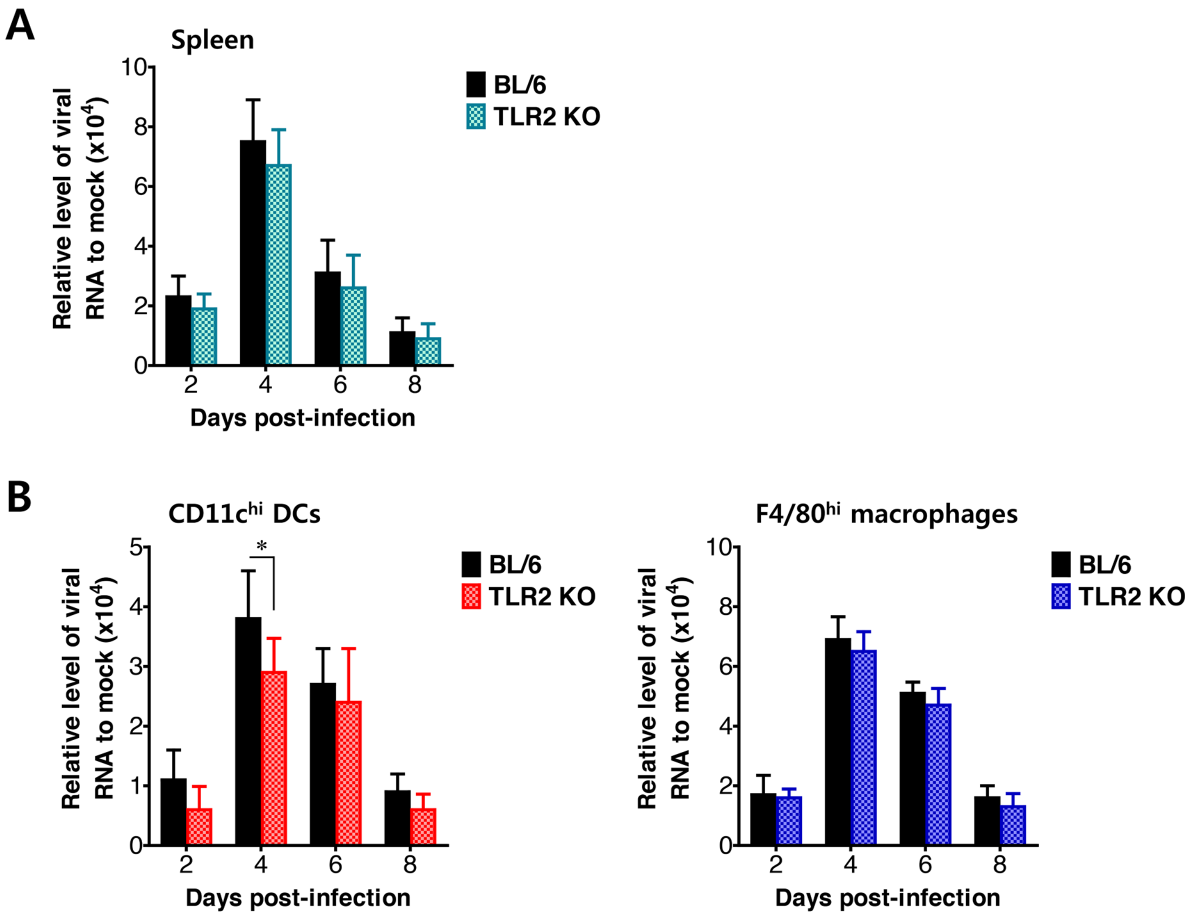

Supplementary Figure 4: *In vivo* multiplication of DenV in splenic DCs and macrophages of BL/6 and TLR2 KO mice. CD11c^{hi} DCs and F4/80^{hi} macrophages were sorted from the spleen of BL/6 and TLR2 KO mice at the indicated time pi. Total RNAs were extracted from the spleen and sorted cells, and used to determine viral RNA by real-time qRT-PCR. Levels of viral RNA were expressed as relative levels to uninfected samples after normalized with β-actin. Data in graphs denote the average ± SE of levels derived from at least four independent experiments. **(A)** spleen, **(B)** CD11c^{hi} DCs, **(C)** F4/80^{hi} macrophages. *, $p < 0.05$ compared to levels in the indicated group.

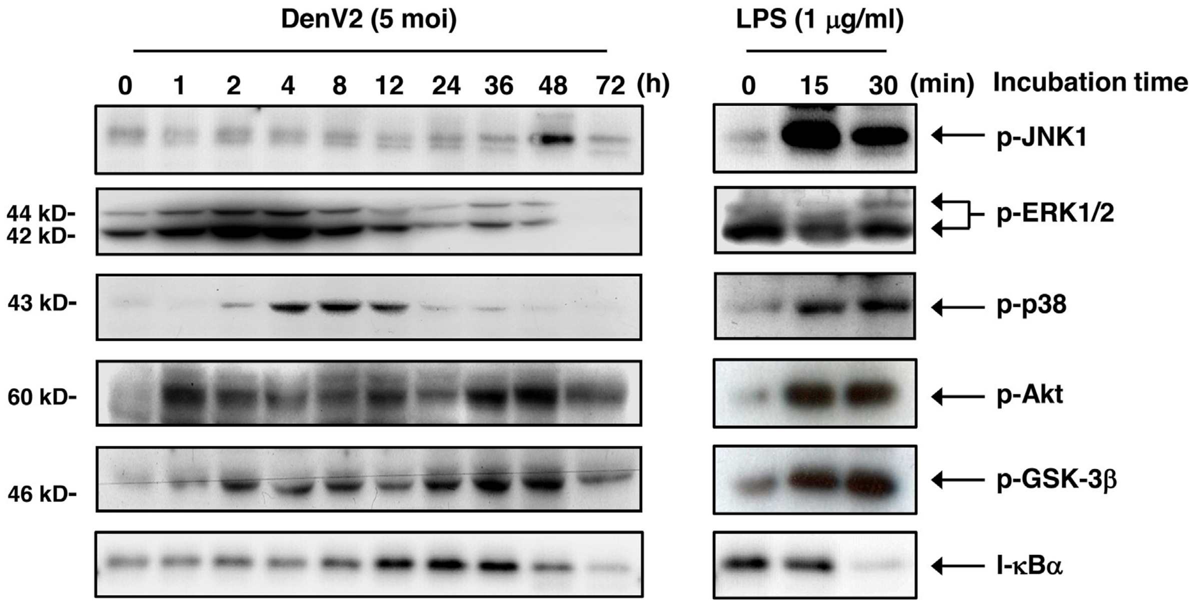

Supplementary Figure 5: Dengue virus infection induces I-κBα degradation and phosphorylation of MAPKs in DCs. DCs (DC2.4) were infected with DenV2 (5.0 moi) for different time periods. Cells were then lysed, separated by SDS-PAGE, and analyzed by western blot to detect unphosphorylated and phosphorylated forms of target proteins using specific Abs. LPS-treated BMDC was used as positive control. Similar results were obtained in three independent experiments.
